# MyD88 oligomer size functions as a physical threshold to trigger IL1R Myddosome signaling

**DOI:** 10.1083/jcb.202012071

**Published:** 2021-05-06

**Authors:** Rafael Deliz-Aguirre, Fakun Cao, Fenja H.U. Gerpott, Nichanok Auevechanichkul, Mariam Chupanova, YeVin Mun, Elke Ziska, Marcus J. Taylor

**Affiliations:** Max Planck Institute for Infection Biology, Berlin, Germany

## Abstract

A recurring feature of innate immune receptor signaling is the self-assembly of signaling proteins into oligomeric complexes. The Myddosome is an oligomeric complex that is required to transmit inflammatory signals from TLR/IL1Rs and consists of MyD88 and IRAK family kinases. However, the molecular basis for how Myddosome proteins self-assemble and regulate intracellular signaling remains poorly understood. Here, we developed a novel assay to analyze the spatiotemporal dynamics of IL1R and Myddosome signaling in live cells. We found that MyD88 oligomerization is inducible and initially reversible. Moreover, the formation of larger, stable oligomers consisting of more than four MyD88s triggers the sequential recruitment of IRAK4 and IRAK1. Notably, genetic knockout of IRAK4 enhanced MyD88 oligomerization, indicating that IRAK4 controls MyD88 oligomer size and growth. MyD88 oligomer size thus functions as a physical threshold to trigger downstream signaling. These results provide a mechanistic basis for how protein oligomerization might function in cell signaling pathways.

## Teaser

Deliz-Aguirre et al. examine the molecular dynamics of interleukin 1 receptor signaling. Receptor sensing of IL-1 triggers the oligomerization of MyD88 and formation of the Myddosome signaling complex. The formation of a MyD88 oligomer of a requisite size serves as a threshold to active downstream signaling.

## Introduction

The innate immune system is a form of host defense that rapidly responds to infection and disease. Central to an innate immune response are diverse germline encoded receptor families that recognize the molecular signals of infection and disease (Brubaker et al., 2015). A unifying property of innate immune receptor signaling pathways is the self-assembly of signaling proteins into large macromolecular complexes (Kagan et al., 2014). Structural and molecular characterization revealed that these macromolecular assemblies are oligomeric, with signaling effectors able to polymerize into structurally defined complexes (Wu, 2013), and are collectively referred to as supramolecular organizing centers (SMOCs; Kagan et al., 2014).

Unlike receptor tyrosine kinases or G-protein–coupled receptors, many innate immune receptors are not enzymatically active or directly linked to secondary messengers, such as calcium or cAMP (Wu, 2013). Furthermore, the signaling effectors that bind to activated receptors and self-assemble into SMOCs do not contain enzymatic activity. Therefore, innate immune receptors such as inflammasome receptors, TNF receptors, Toll-like receptors (TLRs), and interleukin 1 (IL1) receptors (IL1Rs) cannot simply transduce signals by up-regulating enzymatic activity (Wu, 2013; Kagan et al., 2014). Thus, a model for SMOC signaling is that these macromolecular complexes are inducible platforms that form on demand and activate signaling by concentrating and activating downstream enzymatic effectors (Tan and Kagan, 2019). However, how is receptor-triggered oligomerization controlled to accurately and rapidly transduce a signal? How large must a SMOC oligomer be, and how long must it persist to achieve downstream signaling?

One such SMOC, the Myddosome, is a macromolecular complex consisting of helical oligomers of MyD88, and kinases of the IL1R-associated kinase (IRAK) family (Motshwene et al., 2009; Lin et al., 2010). The Myddosome mediates signaling from the TLR/IL1R superfamily (Gay et al., 2014). Members of the TLR/IL1R superfamily are critical mediators of a protective innate immune response and are characterized by the presence of a cytoplasmic Toll-IL1R (TIR) domain (Gay et al., 2014). IL1Rs respond to inflammatory cytokines of the IL1 family (Dinarello, 2018), whereas TLRs respond to microbial- and viral-associated molecules (Fitzgerald and Kagan, 2020). The Myddosome biochemically interacts with activated TLR/IL1Rs via the TIR domain–containing cytoplasmic adapter MyD88 (Nimma et al., 2017). MyD88 has no intrinsic enzymatic activity and contains an N-terminal death domain (DD) and a C-terminal TIR domain (Hardiman et al., 1996). MyD88 biochemically interacts with TLR/IL1Rs via heterotypic TIR domain interactions (Medzhitov et al., 1998), and self-assembles via its DD into helical oligomers (Lin et al., 2010). It is these helical MyD88 oligomers that couple to enzymatic activity by coassembling with the DD-containing Ser/Thr kinases of the IRAK family.

MyD88 oligomerization is thought to be triggered by TLR/IL1R ligand binding and receptor dimerization. This is believed to stimulate the recruitment and coassembly of IRAKs at the plasma membrane. Structural studies on purified Myddosomes revealed a hierarchical order of stacked DD oligomers consisting of six MyD88s, followed by four IRAK4s and four IRAK2s (Lin et al., 2010). This organization suggests a sequential order of assembly, i.e., MyD88 polymerization triggers the recruitment of IRAK4 followed by IRAK2 (or the functionally redundant IRAK1; Kawagoe et al., 2008). However, purified MyD88 DDs and full-length protein can polymerize into helical open-ended filaments in vitro (Moncrieffe et al., 2020; Motshwene et al., 2009; O’Carroll et al., 2018). Therefore, it is not clear how the coassembly of MyD88 and IRAKs is controlled at a precise time and place within live cells.

Visualizing the spatiotemporal dynamics of Myddosome assembly in living cells could unveil hitherto hidden mechanisms of TLR/IL1R signal transduction. Live-cell analysis of Myddosome dynamics has been limited to individual proteins such as MyD88 (Latty et al., 2018). This has made it difficult to determine how the multiple proteins required for TLR/IL1R signaling are temporally coordinated and to identify precise stages in Myddosome assembly. Here, we developed a live imaging approach to directly visualize Myddosome formation in response to IL1β stimulation in EL4 cells. We engineered precise fluorescent protein fusions of Myddosome proteins at endogenous gene loci using CRISPR/Cas9. By simultaneously imaging and quantifying multiple signaling reactions, we discovered that the formation of larger MyD88 oligomers functions as a signaling threshold to trigger IRAK kinase recruitment to the cell surface, and that IRAK4 regulates MyD88 oligomerization. Collectively, these results highlight how protein oligomerization can transduce biochemical signals from activated IL1Rs. This provides a conceptual framework for understanding SMOC assembly in diverse innate immune receptor signaling pathways.

## Results

### Membrane-tethered IL1β triggers the relocalization of MyD88 to the cell surface and nuclear translocation of RelA

**Figure S1.**
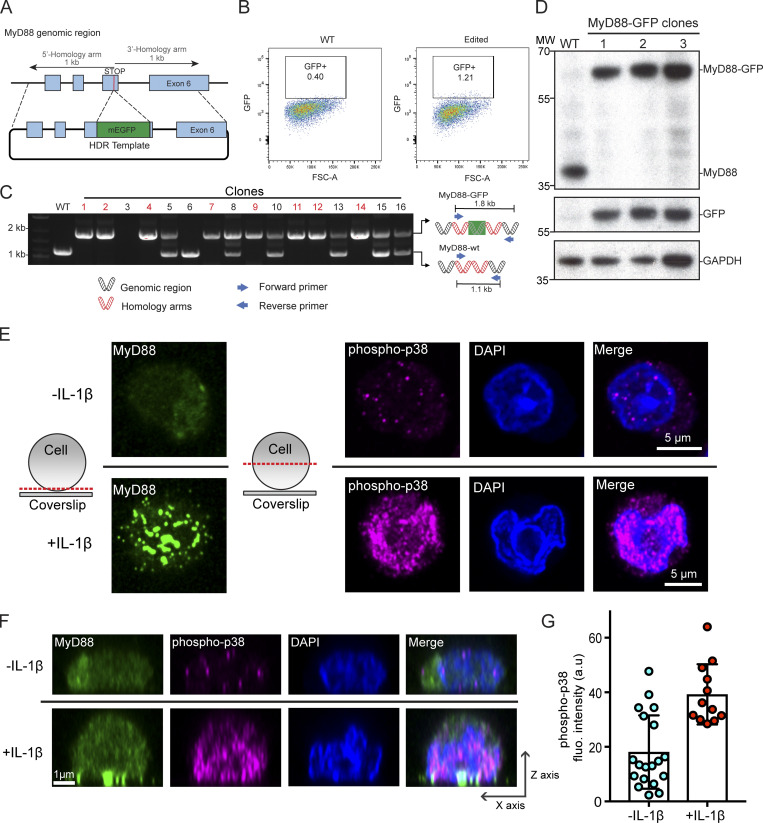
**CRISPR/Cas9 gene editing of the MyD88 gene locus with mEGFP and p38 signaling in EL4-MyD88-GFP cells. (A–D)** Workflow and validation for CRISPR/Cas9 editing of the MyD88 gene locus with mEGFP. **(A)** Schematic of the MyD88 gene locus and HDR template designed to insert a mEGFP open reading frame immediately upstream of the stop codon. **(B)** FACS results of WT and CRISPR/Cas9-engineered MyD88-GFP EL4 cells. Overlaid box indicates a sorting gate to select EL4-MyD88-GFP cells. **(C)** PCR screening of CRISPR/Cas9 editing to select homozygous edited cell clones. Schematic shows the primer design for PCR amplification of genomic DNA to detect WT, heterozygous, and homozygous edited EL4 cells. Homozygous MyD88-GFP edited cell clones are labeled in red. Three homozygous clones were retained for Western blot analysis. See Fig. S6 for uncropped blots. **(D)** Western blot analysis of three MyD88-GFP clones. Blots were probed with anti-MyD88, then stripped and reprobed with anti-GFP. **(E)** To assess the level of MAPK pathway activation, EL4 cells were fixed (60 min after SLB contact) and stained for MyD88-GFP (green) and phospho-p38 (magenta); DAPI staining of nuclei (blue). Cells were imaged with confocal microscopy. Schematic shows the position of the confocal micrograph slice. Only cells in contact with SLB functionalized with IL1β had increased phospho-p38 nuclear staining intensity. Scale bar, 5 mm. **(F)** Reconstructed axial view of cells shown in E showing the localization of MyD88 to the cell–SLB contact zone and phospho-p38 staining under IL1β stimulation. Scale bar, 1 mm. **(G)** Quantification of phospho-p38 staining intensity. Mean ± SD from *n* = 12 (with IL1β) and 20 cells (without IL1β). fluo, fluorescence; FSA-A, forward scatter area; MW, molecular weight.

We used CRISPR/Cas9 gene editing to generate monoclonal cell lines with MyD88 tagged at the endogenous gene locus with a C-terminus monomeric enhanced GFP (Fig. S1, A–D; and Fig. S6). We performed gene editing in the mouse lymphoma T cell line EL4.NOB1 (referred to as EL4 cells; see Materials and methods). We selected EL4 cells because they are highly responsive to IL1 and have previously been used to study IL1 signaling (O’Neill et al., 1990; Bird et al., 1988). Our knockin strategy enabled us to limit overexpression artifacts and quantitatively compare cells and measurements across experiments.

**Figure 1.**
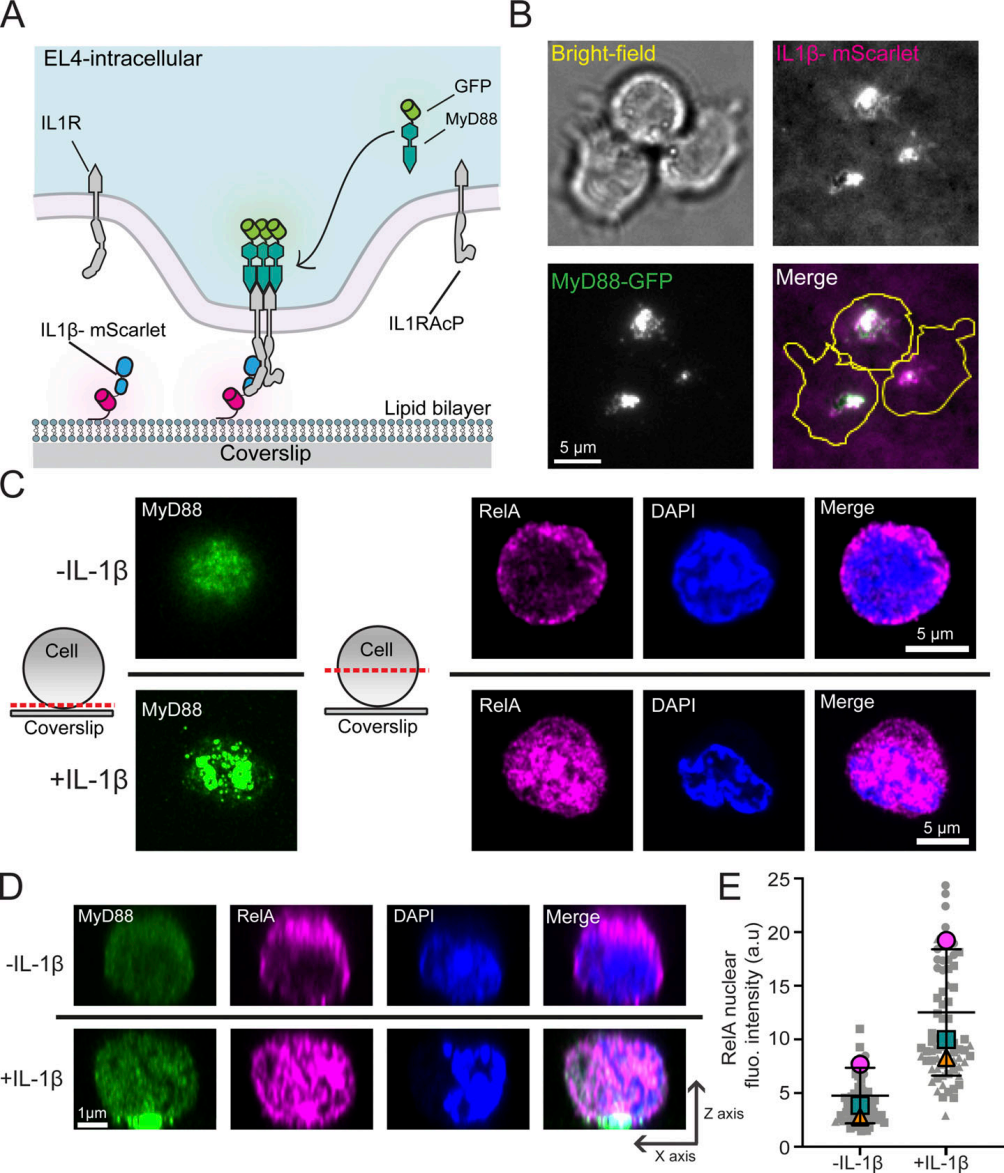
**Membrane-tethered IL1β triggers the relocalization of MyD88 to the cell surface and nuclear translocation of RelA. (A)** The schematic of a SLB functionalized with IL1β labeled with mScarlet. **(B)** TIRF and brightfield microscopy images of EL4 cells expressing MyD88-GFP after landing on a SLB functionalized with IL1β-mScarlet. Clusters of IL1β-mScarlet formed at the cell–SLB interface. MyD88-GFP was recruited to clusters of IL1β-mScarlet. **(C and D)** EL4 cells were fixed (60 min after SLB contact) and stained for MyD88-GFP (green) and RelA (magenta), with DAPI staining of nuclei (blue). Cells were imaged with confocal microscopy. **(C)** Schematic shows the position of the confocal micrograph slice. Cells in contact with IL1β-functionalized SLBs relocalize RelA to the nucleus. **(D)** Reconstructed axial view of cells shown in C, showing the localization of MyD88 to the cell–SLB contact zone and RelA to cell nucleus under IL1β stimulation. **(E)** Quantification of RelA nuclear staining intensity. Bar represents mean ± SEM from *n* = 3 experimental replicates. Scatter plot symbols represent independent replicates, smaller gray symbols represent single-cell measurements, and superimposed larger symbols represent the averages from experimental replicates. fluo., fluorescence.

To stimulate IL1R signaling and Myddosome formation in live EL4 cells, we developed planar supported lipid bilayers (SLBs) functionalized with freely diffusing IL1β (Fig. 1 A). Since IL1R signals in response to soluble and membrane-bound isoforms of IL1 (Kaplanski et al., 1994), we reasoned that IL1 tethered to SLBs reconstituted the IL1R signaling at cell–cell contact sites. Finally, the planar geometry of SLBs can be combined with total internal reflection (TIRF) microscopy to directly visualize signaling reactions at the cell surface (Biswas and Groves, 2019).

We pipetted EL4-MyD88-GFP cells into chambers containing IL1β-mScarlet–labeled SLBs (Fig. 1 A). Cells were allowed to settle for 20 min before being imaged using TIRF and bright-field microscopy. We observed that IL1β-mScarlet was clustered at the EL4 cell–bilayer interface. MyD88-GFP was recruited to this interface and assembled into large fluorescent clusters. These macromolecular clusters colocalized with the IL1β-mScarlet clusters (Fig. 1 B).

Activation of IL1R triggers the stimulation of nuclear factor–κB (NF-κB) protein complex and the translocation of the p65-RelA subunit into the nucleus. We analyzed the subcellular distribution of RelA in EL4-MyD88-GFP cells incubated with IL1β-labeled SLBs for 60 min before being fixed and stained with anti-RelA. The cellular volume was then imaged with confocal microscopy (Fig. 1, C and D). Cells incubated with IL1β-labeled SLBs clustered MyD88-GFP at the cell-bilayer interface. The accumulation of MyD88-GFP was associated with the localization of RelA to the nucleus (Fig. 1, C and D). In EL4 cells incubated with unlabeled SLBs, MyD88-GFP remained diffuse throughout the cytoplasm, and RelA was excluded from the nucleus, thereby resulting in lower nuclear staining intensities (Fig. 1 E). Similar to NF-κB activation, we found that EL4 cells incubated with IL1β-labeled SLBs had increased levels of phospho-p38 (Fig. S1, E–G). Thus, IL1β tethered to supported membranes can activate NF-κB, MAPK p38 signaling, and the relocalization of MyD88 to the cell surface.

**Figure S2.**
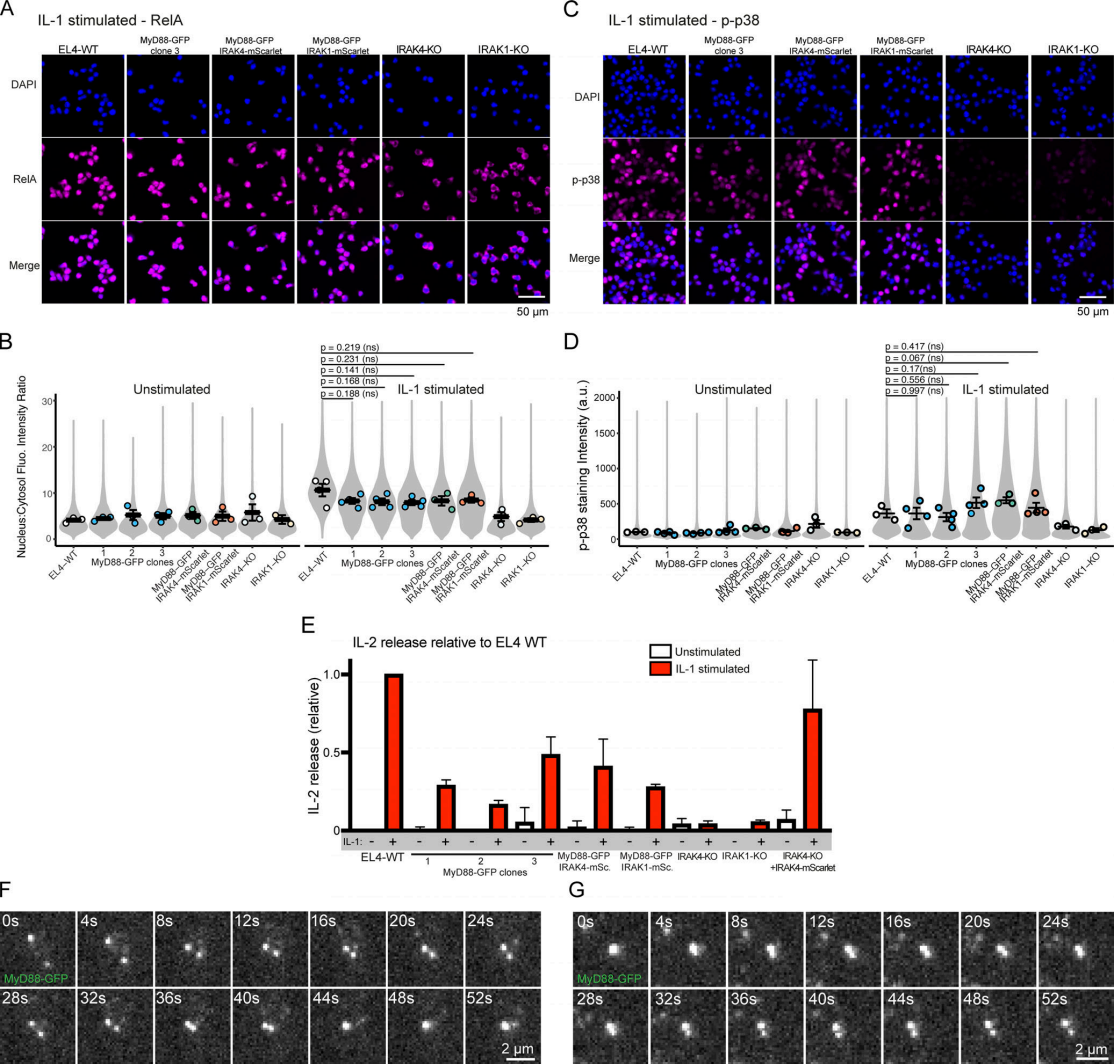
**Quantification of RelA nuclear translocation, MAPK p38 induction, and cytokine release in gene-edited EL4 cells. (A)** RelA translocation to the nucleus in WT and gene-edited EL4 cells. EL4 cell lines (30 min after addition to IL1β-labeled SLBs) were fixed and stained for RelA (magenta); DAPI stained nuclei (blue). Scale bar, 50 µm. **(B)** Quantification of RelA nucleus-to-cytoplasm staining ratio. Violin plots show the single-cell distribution RelA nucleus-to-cytoplasm staining ratio. Colored dots superimposed on violin plots correspond to the average value in the independent experiments (*n* = 3 or 4 experimental replicates per cell line; each replicate encompasses measurements from >2,000 cells. Bars represent mean ± SEM. P values were calculated using a two-tailed unpaired Student's *t* test. **(C)** Phospho-p38 staining intensity in WT and gene-edited EL4 cells. EL4 cell lines (30 min after addition to IL1β-labeled SLBs) were fixed and stained for phospho-p38 (magenta); DAPI stained nuclei (blue). Scale bar, 50 µm. **(D)** Quantification of phospho-p38 staining intensity. Violin plots show the single cell distribution phospho-p38 staining intensity. Colored dots superimposed on violin plots correspond to the average value in the independent experiments (*n* = 3 or 4 experimental replicates per cell line; each replicate encompasses measurements from >6,000 cells). Bars represent mean ± SEM. P values were calculated using a two-tailed unpaired Student's *t* test. **(E)** IL2 release in WT and gene-edited EL4 cells. IL2 release was measured by ELISA 24 h after IL1β stimulation. Values for gene-edited cells shown relative to EL4 WT. Average values calculated from three independent experiments. Bars represent mean ± SEM. **(F and G)** MyD88-GFP puncta can fuse and split. **(F)** Example of two MyD88-GFP puncta undergoing fusion. **(G)** Example of MyD88-GFP puncta undergoing fission. Scale bar, 2 µm. Fluo., fluorescence; p-p38, phospho-p38.

We compared the IL1 signaling response of EL4 WT and MyD88-GFP CRISPR/Cas9 gene-edited cell lines (and all gene-edited cell lines generated; see below; Fig. S2, A–E). After a 30-min incubation with IL1β-labeled SLBs, we found that MyD88-GFP and WT EL4 cells had no statistical difference in NF-κB RelA nuclear translocation and phospho-p38 MAPK staining intensity (Fig. S2, A–D). IL2 expression in EL4 cells is induced by IL1 stimulation and NF-κB activation (Knop et al., 1998). We found that all MyD88-GFP clones released IL2 in response to IL1β stimulation (although IL2 release was reduced relative to WT EL4 cells; Fig. S2 E). We conclude that tagging MyD88 with GFP does not abolish IL1R-signaling responses.

### MyD88-GFP puncta form at the cell surface and colocalize with clusters of IL1R-bound IL1β

**Figure 2.**
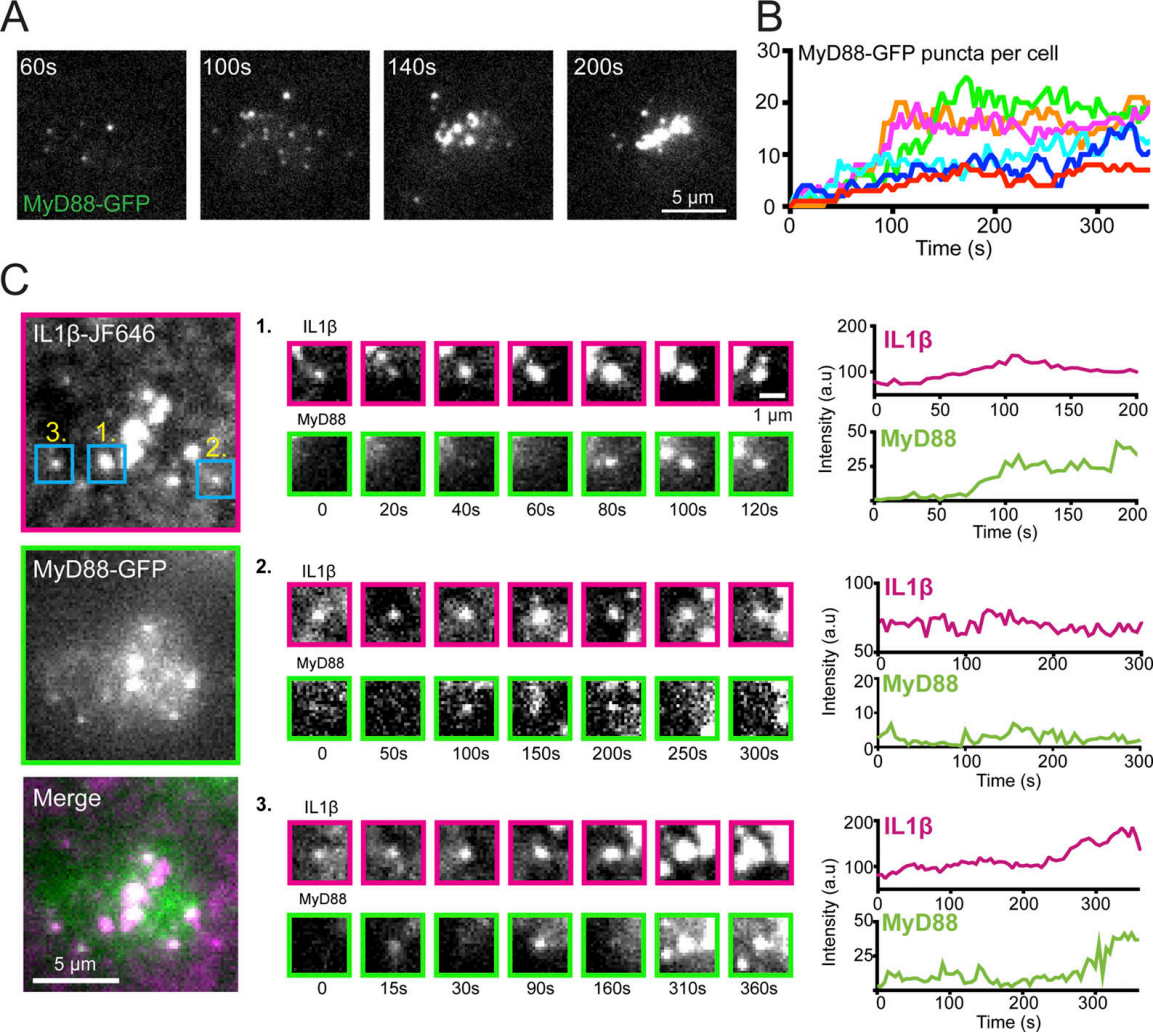
**MyD88-GFP puncta assembly at clusters of receptor-bound IL1β. (A)** Time-lapse TIRF images of MyD88-GFP showing the formation and coalescence of MyD88-GFP puncta at the cell surface. **(B)** Formation of MyD88-GFP puncta in individual cells (colors). Time = 0 is defined as the point of image acquisition. **(C)** TIRF images of IL1β-JF646 and MyD88-GFP showing Myddosome formation at clusters of IL1β-bound IL1R. Regions of interest (blue boxes) show three IL1β-receptor clusters (labeled 1–3). Fluorescence intensity time series from the IL1β-JF646 and MyD88-GFP of the three IL1β clusters are shown on the right.

We examined the temporal dynamics of MyD88-GFP in EL4 cells stimulated with IL1β. EL4-MyD88-GFP cells were applied to SLBs and imaged using TIRF microscopy (Fig. 2 A). We observed the formation of mobile MyD88-GFP puncta at the cell–bilayer interface that, after several minutes, coalesced into larger clusters (Fig. 2 A). MyD88-GFP puncta underwent both fusion and fission (Fig. S2, F and G). We quantified the formation of MyD88-GFP puncta as a function of time after cell landing. Within 5 min of contacting the IL1β-functionalized SLBs, EL4 cells rapidly formed many (5–25) MyD88-GFP puncta (Fig. 2 B).

We investigated the recruitment of MyD88 to IL1β-IL1R complexes. To observe IL1β-IL1R engagement, we engineered a Halo-tag version of IL1β and labeled it with the photostable organic fluorophore JF649. Using two-color TIRF microscopy, we imaged cellular MyD88-GFP and IL1β-JF649 on the SLBs. When EL4 cells contacted the SLBs, we observed the homogeneous distribution of IL1β-JF646 reorganized into microclusters (Fig. 2 C and Video 1). IL1β microclusters increased in fluorescence intensity over time, indicating the addition of newly IL1R-bound IL1β-JF649. IL1β microclusters appeared as diffraction-limited clusters, but over time coalesced into nondiffraction patch-like structures. MyD88 spots formed at clusters of IL1β at the cell–bilayer interface. The formation of IL1β clusters preceded the assembly of MyD88 into puncta (Fig. 2 C). From these data, we conclude that IL1β binding to IL1R stimulates the recruitment of MyD88 to the plasma membrane.

**Video 1.** **IL1β tethered to a supported lipid membrane forms clusters that recruit MyD88-GFP (related to ****Fig. 2****).** This video shows an EL4 cell expressing MyD88-GFP (middle, green channel in merge) interacting with a SLB functionalized with IL1β-JF646 (left, magenta channel in merge). The video illustrates that the IL1β clustering at the cell-supported membrane interface precedes the recruitment and formation of MyD88-GFP puncta at the cell surface. Same cell as shown in Fig. 2 C. Scale bar, 5 µm.

We observed that MyD88 had heterogeneous recruitment dynamics to clusters of IL1β-bound IL1R. We observed the formation of stable MyD88 puncta (“stable” defined as persisting for >1 min; Fig. 2 C, example 1). The stable MyD88 puncta appeared ∼1–2 min after the formation of an IL1β cluster. We also observed the formation of transient MyD88 foci at the cell surface. These transient foci were dimmer than the stable MyD88 puncta and did not increase in fluorescence intensity (Fig. 2 C, example 2). In some cases, we observed both types of dynamics at the same clusters of IL1R-bound IL1β. In these instances, the transient MyD88-GFP foci preceded the formation of a stable MyD88 punctum (Fig. 2 C, example 3).

### MyD88-GFP forms transient and stable macromolecular assemblies

**Figure 3.**
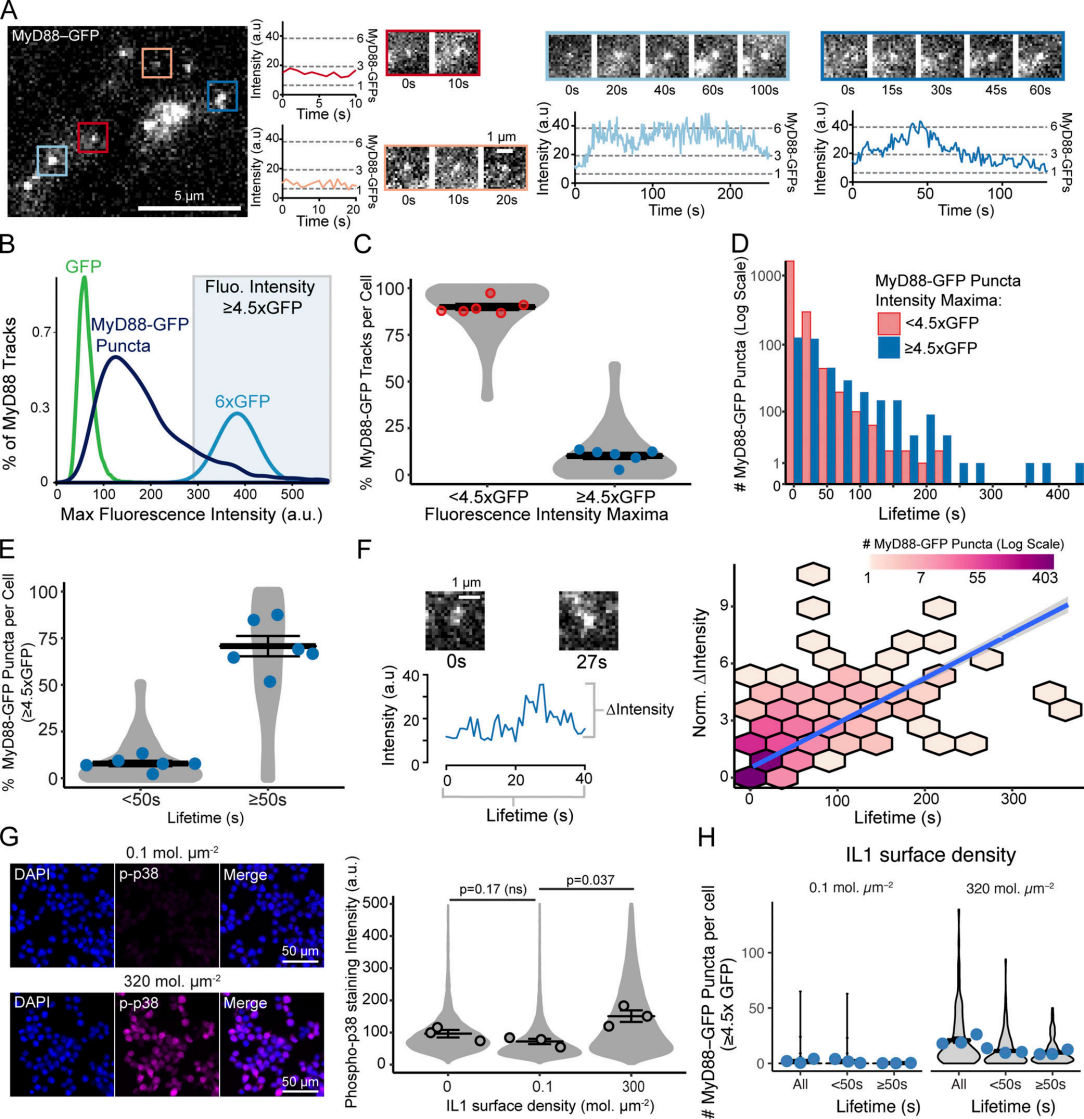
**Analysis of MyD88-GFP puncta dynamics, lifetime, and size. (A)** TIRF images of MyD88-GFP in an EL4 cell landing on an IL1β-functionalized SLB. Overlaid colored boxes highlight examples of individual MyD88-GFP puncta. Red and orange ROI show the fluorescence intensity time series (left) and TIRF images (right) from Myddosomes that are short-lived (<50 s) and dim (<3× GFP average intensity). Blue regions of interest show fluorescence intensity time series (bottom) and TIRF images (top) from two example MyD88-GFP puncta that grow in intensity and have a long lifetime (≥50 s). The dashed gray lines on intensity plots mark the quantal MyD88-GFP fluorescence intensities, estimated from single GFP fluorophores. **(B) **Density plot of the maximum fluorescent intensity of MyD88-GFP puncta (dark blue, *n* = 2,422 tracked MyD88-GFP particles from 14 cells) compared with single molecules of GFP (green, *n* = 397 GFP particles) and estimated intensity distribution of a 6× GFP multimer (light blue). Shaded blue region designates intensity values >4.5× GFP. **(C)** Quantification of the proportion (%) of MyD88-GFP puncta per cell that have a maximum fluorescence intensity <4.5× GFP or ≥4.5× GFP. Violin plots show the distribution of the cell data. Data points superimposed on violin plots are the averages of independent experiments. Bars represent mean ± SEM (*n* = 6 experimental replicates, with 6–24 cells measured per replicate). **(D) **Distribution of MyD88-GFP puncta lifetimes. Myddosomes were classified by maximum fluorescence intensity <4.5× or ≥4.5× GFP (*n* = 2,037 <4.5× GFP versus *n* = 385 ≥4.5× GFP tracked MyD88-GFP puncta combined from 14 cells). **(E)** Quantification of the proportion (%) per cell of MyD88-GFP puncta with an intensity maximum ≥4.5× GFP categorized by lifetimes <50 s or ≥50 s. Violin plots show the distribution of the cell data. Data points superimposed on the violin plots are the averages from independent experiments. Bars represent mean ± SEM (*n* = 6 experimental replicates, with 6–24 cells measured per replicate). **(F) **Correlation between growth in intensity and lifetime of MyD88-GFP puncta. Left: TIRF images and intensity trace of a representative MyD88-GFP puncta. The change in intensity calculated as maximum intensity subtracted by the initial intensity. Right: 2D histogram of MyD88-GFP puncta lifetime versus changed in fluorescent intensity. Linear fit is shown as a blue line with the 95% CI shown in gray (Spearman’s rank correlation coefficient *R* = 0.59, P < 0.001, *n* = 1,763 MyD88 tracks, combined from 14 cells). **(G) **Activation of phospho-p38 at different IL1 ligand densities on SLBs**.** Left: Phospho-p38 staining on SLB labeled with 0.1 and 300 IL1β per square micrometer. EL4 cell lines (30 min after addition to IL1β-labeled SLBs) were fixed and stained for phospho-p38 (magenta); DAPI stained nuclei (blue). Right: Quantification of phospho-p38 staining. Violin plots show the single-cell distribution of staining intensities. Data points superimposed on the violin plots are the averages from independent experiments (*n* = 3 experimental replicates, with >6,000 cells measured per replicate). Bars represent mean ± SEM. P values were calculated using a two-tailed unpaired Student's *t* test. **(H)** Quantification of the number of MyD88-GFP puncta per cell that assemble with intensity maxima ≥4.5× GFP on SLB labeled with high and low IL1 densities. Violin plots show the single-cell distribution of the total number of MyD88-GFP puncta per cell and those with lifetimes <50 s or ≥50 s. Data points superimposed on the violin plots are the averages from independent experiments. Bars represent mean ± SEM (*n* = 3 experimental replicates, with 6–24 cells measured per replicate). P values were calculated using an unpaired Student's *t* test. fluo., fluorescence; Max, maximum; Norm., normalized.

We used automated particle tracking to obtain an unbiased dataset of MyD88 assemblies and then analyzed their lifetimes and intensities (Video 2). To estimate the copy number of MyD88-GFP within puncta, we calibrated our TIRF setup using purified GFP. Congruent with our previous observation (Fig. 2 C), we observed two classes of MyD88-GFP puncta at the cell surface with distinct lifetimes and intensity traces. The first class contained the transient foci that showed a minimal increase in fluorescent intensity. The second class contained foci that increased in fluorescent intensity becoming bright, stable MyD88-GFP puncta (Fig. 3 A, see example of each dynamic; and Video 2). We observed that MyD88-GFP foci corresponding to the first class had fluorescent intensities corresponding to 1–3× the mean intensity of GFP. MyD88 puncta that belonged to the second class had an initial fluorescence intensity equivalent to 1–3× GFP. In contrast to the shorter-lived MyD88 assemblies, these structures increased to a fluorescent intensity in a manner consistent with >6× GFP mean intensity (Fig. 3 A, dashed lines on the intensity time traces).

**Video 2.** **Single cell analysis of MyD88-GFP puncta dynamics (related to ****Fig. 3****).** The video shows an EL4 cell expressing MyD88-GFP imaged using TIRF microscopy, and illustrates MyD88 puncta tracking and analysis of MyD88-GFP puncta for a single cell. Analysis is updated as the video progresses. Panels in the video correspond to the panels in Fig. 3. **(A)** Video of EL4 MyD88-GFP imaged under TIRF microscopy with a time interval of 1 s. Overlay of particle track trajectories. Particle trajectory color-coded according to whether the maximum intensity is ≥4.5× GFP (blue) or <4.5× GFP (red). **(B)** Density plot of the maximum fluorescence intensity of tracked MyD88-GFP puncta from the cell in A (dark blue curve). Intensity distribution of GFP and 6× GFP multimer (green and light blue, respectively) is shown for comparison. Blue background shade indicates ≥4.5× GFP. **(C)** Proportion (%) of MyD88-GFP puncta with a maximum intensity <4.5× GFP (red) or ≥4.5× (blue). **(D)** Lifetime distribution of MyD88-GFP puncta. MyD88-GFP lifetime histogram for MyD88-GFP puncta with a maximum intensity of <4.5× GFP (red) or ≥4.5× GFP (blue). The puncta count is shown in log scale. **(E)** Proportion (%) of MyD88-GFP puncta with a maximum intensity of ≥4.5× GFP with lifetimes of <50 s or ≥50 s. **(F)** Correlation between lifetime and intensity growth of MyD88-GFP puncta. 2D histogram of MyD88-GFP puncta lifetime by change in fluorescent intensity. The linear regression line is shown in blue with 95% CIs in gray. The MyD88-GFP puncta count is shown in log scale.

**Figure S3.**
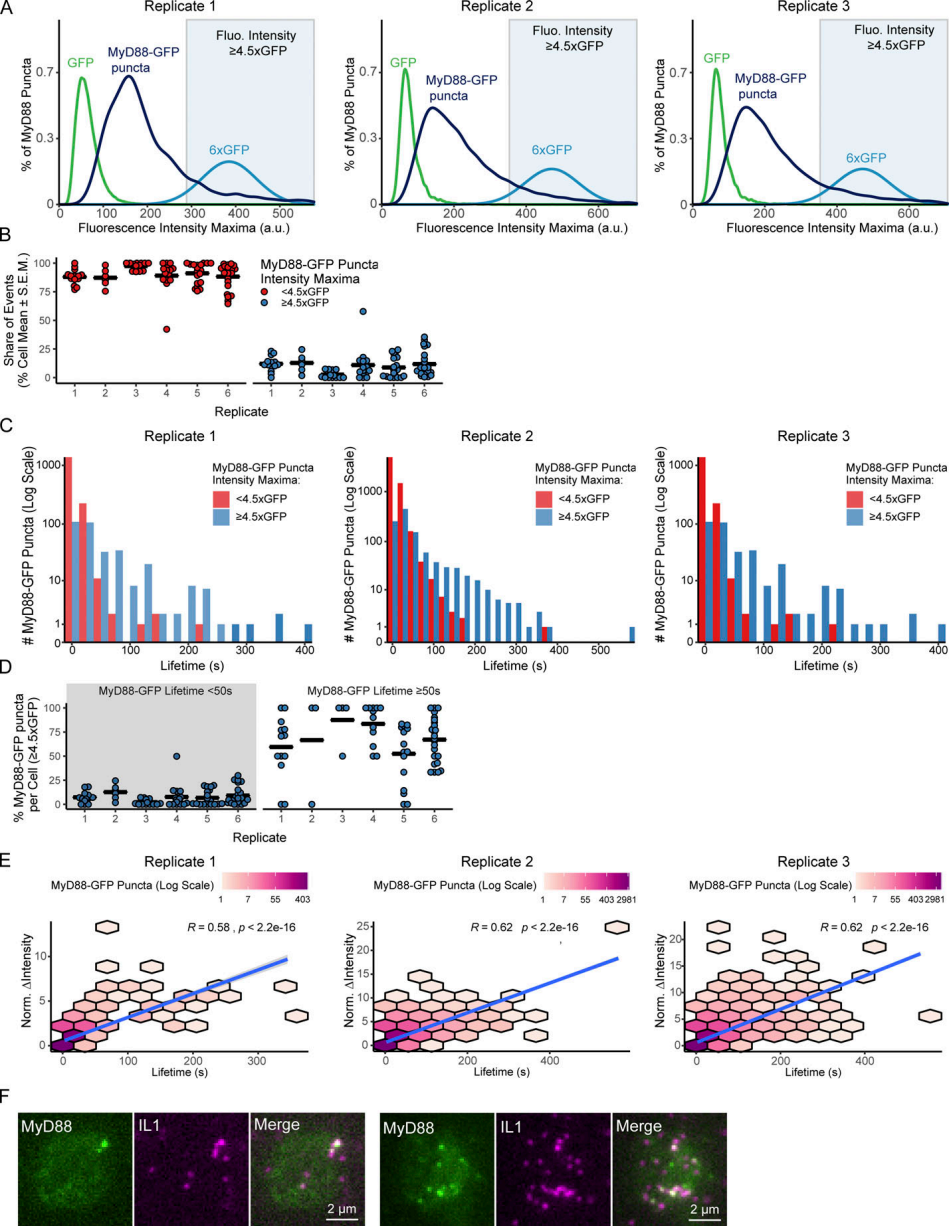
**Analysis of MyD88-GFP puncta size, lifetime, and correlation analysis from biological replicates of ****Fig. 3****. (A)** Size distribution of MyD88-GFP puncta from additional experimental replicates. Density plot of the maximum fluorescence intensity of MyD88-GFP puncta (dark blue; replicate 1, *n* = 1,952 puncta from 16 cells; replicate 2, *n* = 7,637 puncta from 19 cells; replicate 3, *n* = 11,973 puncta from 24 cells). For comparison, we included the intensity distribution of single GFP fluorophores (green; replicate 1, *n* = 298,293 GFP particles; replicate 2, *n* = 7,995 GFP particles; replicate 3, *n* = 7,995 GFP particles). To estimate the distribution of a 6× GFP multimer (light blue), a Gaussian curve was fitted to the 1× GFP intensity distribution (see Materials and methods). Blue background shade indicates ≥4.5× GFP. **(B)** Proportion (%) of the maximum intensity (<4.5× or ≥4.5× GFP) of MyD88-GFP puncta by cell across experimental replicates. Data points are the proportion of the maximum intensity <4.5× GFP (red) or ≥4.5× GFP (blue) by individual cells from six independent experiments. Percentage is the replicate’s proportion of MyD88-GFP puncta with maximum intensity ≥4.5× GFP, n = cell, replicates 1–6: 16% *n* = 6; 16%, *n* = 14; 3%, *n* = 13; 17%, *n* = 16; 13%, *n* = 19; 17%, *n* = 24. **(C)** Lifetime distribution of MyD88-GFP puncta from additional experimental replicates. MyD88-GFP lifetime histogram for MyD88-GFP puncta with maximum intensity <4.5× GFP (red) or ≥4.5× GFP (blue; replicate 1, *n* = 1,616 puncta <4.5× GFP and *n* = 336 ≥4.5× GFP; replicate 2, *n* = 6,553 puncta <4.5× GFP and *n* = 1,084 puncta ≥4.5× GFP; replicate 3, *n* = 9,913 puncta <4.5× GFP, and *n* = 2,060 puncta ≥4.5× GFP). Puncta count is in log scale. **(D)** Proportion (%) of the lifetimes (<50 s or ≥50 s) that are bright MyD88-GFP puncta (≥4.5× GFP) in individual cells across experimental replicates. Data points are the proportion of individual cells from independent experimental replicates. Percentage of MyD88-GFP puncta with a maximum intensity of ≥4.5× MyD88 that are <50 s (n = cells, from replicate 1–6): 7%, *n* = 14; 13%, *n* = 6; 2%, *n* = 13; 8%, *n* = 16; 7%, *n* = 19; 9%, *n* = 24). Percentage of MyD88-GFP puncta with a maximum intensity of ≥4.5×x GFP that have lifetimes ≥50 s (n = cells, replicate 1–6): 59%, *n* = 14; 67%, *n* = 6; 88%, *n* = 13; 83%, *n* = 16; 52%, *n* = 19; 67%, *n* = 24). Long-lived events are more likely to be brighter. Bars in B and D represent the replicate mean. **(E)** Correlation between lifetime and intensity growth of MyD88-GFP puncta from additional experimental replicates. 2D histogram of MyD88-GFP puncta lifetime by change in fluorescence intensity (calculated as maximum intensity minus starting intensity). MyD88-GFP puncta with longer lifetimes have a greater increase in fluorescence intensity. Linear regression line is shown in blue with a 95% CI in gray. There is a statistically significant strong positive correlation between lifetime and growth (*n* = puncta, replicate 1–3: *R* = 0.58, P < 0.001, *n* = 1,952; *R* = 0.62, P < 0.001, *n* = 7,637; *R* = 0.62, P < 0.001, *n* = 11,973). Correlations are Spearman’s rank correlation coefficient. Puncta count is shown in log scale. **(F)** TIRF images of IL1β-JF646 and MyD88-GFP showing MyD88 puncta formation at a membrane density 0.1 IL1β per square micrometer. Fluo, fluorescence; Norm., normalized.

We systematically examined the distribution of MyD88 oligomer sizes. We plotted the distribution of maximum intensities of tracked MyD88 particles and compared this distribution to the fluorescent intensity of single GFP fluorophores. Given that the Myddosome crystal structure contains 6× MyD88s (Lin et al., 2010), we also estimated the fluorescent intensity distribution for a particle containing 6× GFP molecules (Fig. 3 B; see Materials and methods). The MyD88-GFP puncta distribution suggested a broad size distribution of MyD88 oligomer sizes (Fig. 3 B; also see Fig. S3 A). A minority of MyD88 puncta had a fluorescence intensity equivalent to 6× multimers. In contrast, the majority of MyD88 puncta consisted of ∼2–3 MyD88 monomers.

We devised a quantitative classification of MyD88 puncta size (Fig. 3 B). Based on the distribution of single GFP fluorophores, we used a maximum intensity threshold of 4.5× GFP to classify puncta as large MyD88 oligomers (e.g., >4 MyD88s). We subsequently analyzed the proportion of MyD88 puncta with a maximum intensity of <4.5× or ≥4.5× GFP, i.e., defined as small or large MyD88 assemblies, respectively. We applied this classification metric to all particle-tracked MyD88-GFP puncta detected within single EL4 cells (see Video 2). We found that on average, <14% of MyD88 puncta had a maximum intensity of ≥4.5× (Fig. 3 C and Fig. S3 B). The majority of MyD88 puncta had intensities of <4.5× GFP (86 ± 2% of MyD88-GFP puncta per cell, mean ± SEM; Fig. 3 C and Fig. S3 B). Many of these small MyD88 oligomers had short lifetimes of 3–10 s (Fig. 3 D and Fig. S3 C). In contrast, larger MyD88 oligomers had longer lifetimes and could persist for >100 s (Fig. 3 D).

We classified MyD88 puncta as short- or long-lived (defined as <50 s or ≥50 s) and analyzed the proportion of large assemblies per cell in each lifetime category. We found that 8% of MyD88 puncta per cell with lifetimes <50 s were large assemblies. In contrast, 69% of MyD88 puncta per cell with lifetimes ≥50 s were large oligomers (Fig. 3 E, and Fig. S3 D). Thus, long-lived and stable MyD88 puncta tend to be larger assemblies.

We hypothesized that if MyD88 oligomerization is inducible, the stable larger MyD88 puncta would start as small assemblies and then grow in intensity. Small, unstable MyD88 oligomers that fail to grow would rapidly disassemble. Consistent with this hypothesis, we found that growth in fluorescent intensity correlated with a longer lifetime of MyD88-GFP puncta at the cell surface (Spearman’s rank correlation coefficient, *R* = 0.59; Fig. 3 F and Fig. S3 E). Thus, we find that MyD88-GFP puncta are oligomers of MyD88 nucleating in response to IL1R activation. The majority of these oligomers are transient and small (e.g., consisting of 2–3 MyD88 monomers; Fig. 3 B). However, a portion of the nucleated oligomers recruit additional MyD88 and grow to become stable oligomers that can persist at the cell surface for lifetimes of ≥50 s.

We tested whether the formation of large, stable MyD88-GFP puncta correlated with activation of p38 MAPK signaling. We compared two IL1β-functionalized SLBs labeled with low and high IL1β densities. After a 30-min incubation, the low IL1β density had phospho-p38 staining intensities equivalent to an unlabeled SLB, while the high IL1β membrane density activated a p38 response (Fig. 3 G). Despite no detectable p38 signaling, we still detected the recruitment of MyD88-GFP to small IL1β-JF646 clusters at this low IL1β ligand density (Fig. S3 F). Analysis of the MyD88 puncta dynamics over 30 min revealed that cells assembled 20.9 ± 2.6 versus 2.2 ± 1 MyD88-GFP ≥4.5× puncta per cell in the high ligand versus low ligand regimen (Fig. 3 H, mean ± SEM). When we analyzed the number of MyD88-GFP puncta ≥4.5× with lifetimes ≥50 s, we found 9.8 ± 1.4 versus 0.2 ± 0.07 per cell in the high ligand and low ligand density, respectively (Fig. 3 H). These data are consistent with p38 MAPK activation in IL1R signaling requiring the formation of large, stable MyD88 oligomers.

### IRAK4 and IRAK1 are recruited to larger and kinetically stable MyD88 oligomers

**Figure S4.**
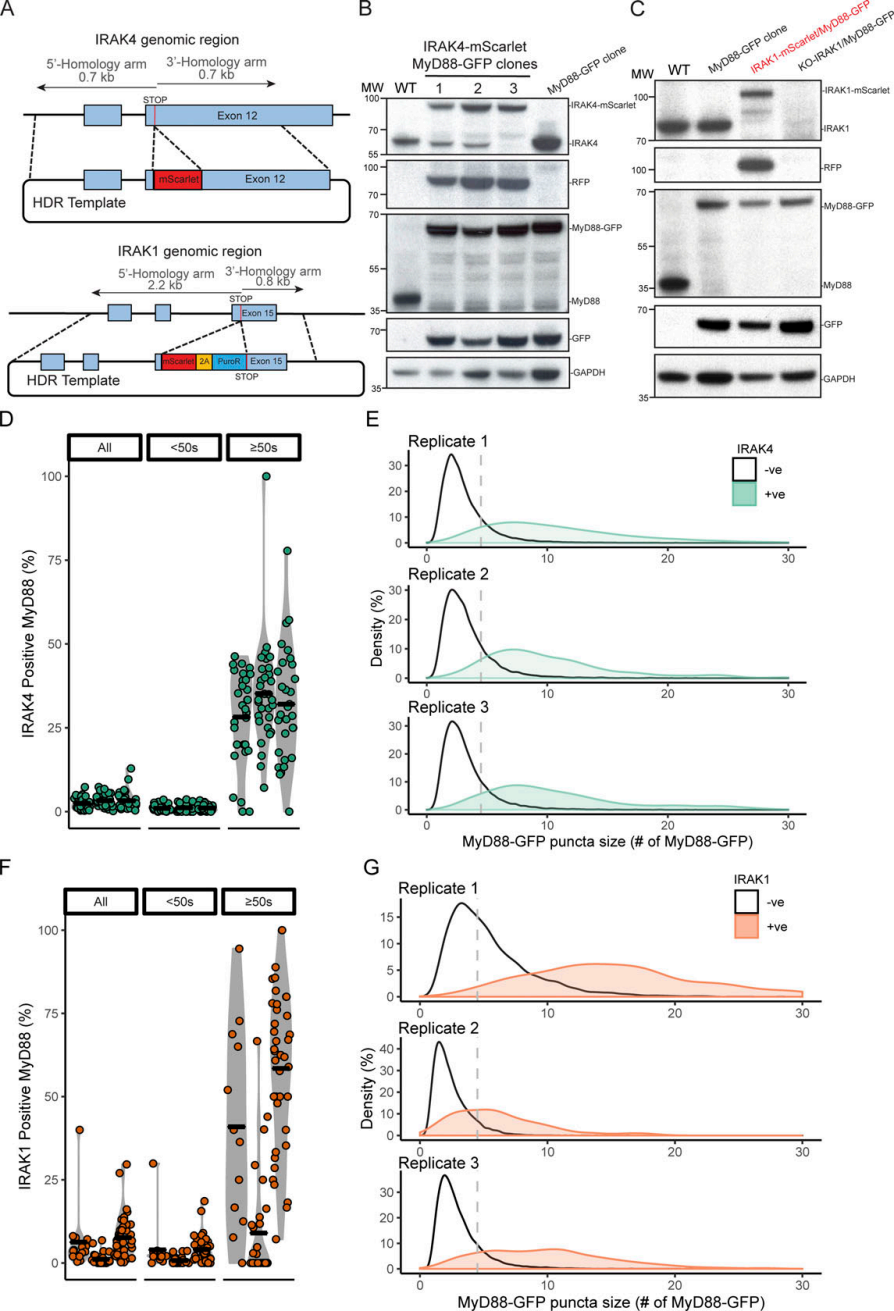
**CRISPR/Cas9 gene editing IRAK4 or IRAK1 gene loci with mScarlet and analysis of MyD88-GFP and IRAK4/1-mScarlet colocalization.** **(A)** Schematic of the IRAK4 (top) and IRAK1 (bottom) gene locus and HDR template designed to insert a mScarlet open reading frame immediately upstream of the stop codon. EL4 cells were electroporated with HDR and gRNA/Cas9 plasmids to simultaneously edit MyD88 and IRAK4 or IRAK1 gene loci. Dual edited cells were selected by FACS and PCR (see Fig. S1, A–D for workflow, and Materials and methods). **(B)** Western blot analysis of three MyD88-GFP/IRAK4-mScarlet–expressing EL4 clones. Lysates were probed with anti-IRAK4, anti-RFP, anti-MyD88, and anti-GFP to confirm editing and insertion of fluorescent protein open reading frames at both gene loci. All data presented in the manuscript were acquired with clone 3. **(C)** Western blot analysis of MyD88-GFP/IRAK1-mScarlet–expressing EL4 clone. Lysates were probed with anti-IRAK4, anti-RFP, anti-MyD88, and anti-GFP to confirm editing and insertion of mScarlet or mEGFP open reading frames at both gene loci. See Figs. S7 and S8 for uncropped blots. **(D–G)** Data shown from clonal MyD88-GFP/IRAK4-mScarlet–expressing EL4 cells (green, D and E) or IRAK1-mScarlet (orange, F and G). **(D and F)** Data points are the proportion of individual cells from independent experiments. Bars are experimental replicate means. **(E and G)** Vertical line in E and G is at 4.5× MyD88-GFP. **(D)** Percentage (%) of MyD88-GFP puncta that colocalizes with IRAK4-mScarlet, combined, and by MyD88-GFP lifetime (<50 s or ≥50 s) per cell across experimental replicates. Violin plot of the percent of MyD88-GFP puncta that is colocalized with IRAK4-mScarlet, combined, and categorized by lifetime (<50 s or ≥50 s). Few tracks recruit IRAK4 (“All”, n = cells, replicates 1–3: 2.4%, *n* = 30; 3.2%, *n* = 31; 3.3%, *n* = 30). It is especially evident in MyD88-GFP puncta that persist for <50 s (“<50 s”, *n* = cells, replicates 1–3: 1.0%, *n* = 30; 1.2%, *n* = 31; 1.1%, *n* = 30). However, MyD88-GFP puncta that persist for ≥50 s are more likely to recruit IRAK4 (“≥50 s”, *n* = cells, replicates 1–3: 28%, *n* = 30; 35%, *n* = 31; 32%, *n* = 30). **(E) **Maximum MyD88-GFP size of IRAK4-mScarlet colocalized and noncolocalized puncta across experimental replicates. Density plot of MyD88-GFP size categorized as colocalized (green) or noncolocalized (black) with IRAK4-GFP. MyD88-GFP puncta colocalized with IRAK4-mScarlet are brighter (mean colocalized versus not colocalized, *n* = puncta, replicates 1–3: 11× MyD88-GFP, *n* = 835 versus 3.1× MyD88-GFP, *n* = 29,072; 10× MyD88-GFP, *n* = 601 versus 3.3× MyD88-GFP, *n* = 17,221; 11.7× MyD88-GFP, *n* = 552 versus 3.3× MyD88-GFP, *n* = 17,854). **(F)** Percentage (%) of MyD88-GFP puncta that colocalize with IRAK1-mScarlet, combined and by MyD88-GFP lifetime (<50 s or ≥50 s) per cell across experimental replicates. Few tracks recruit IRAK1 (“All”, n = cells, replicates 1–3: 6.3%, *n* = 15; 1.5%, *n* = 32; 7.6%, *n* = 40). It is especially evident in MyD88-GFP puncta that persist for <50 s (“<50 s”, *n* = cells, replicates 1–3: 3.9%, *n* = 15; 0.74%, *n* = 32; 4.1%, *n* = 40). However, MyD88 puncta that persist for ≥50 s are more likely to recruit IRAK1 (“≥50 s”, *n* = cells, replicates 1–3: 41%, *n* = 15; 9.0%, *n* = 32; 59%, *n* = 40). **(G) **Maximum MyD88-GFP size of IRAK1-mScarlet colocalized and noncolocalized puncta across experimental replicates. Density plot of MyD88-GFP size categorized as colocalized (orange) or not colocalized (black) with IRAK1-GFP. MyD88-GFP puncta colocalized with IRAK1-mScarlet are brighter (mean colocalized versus not colocalized, *n* = puncta, replicates 1–3: 19× MyD88-GFP, *n* = 291 versus 5.6× MyD88-GFP, *n* = 5,401; 6.2× MyD88-GFP, *n* = 314 versus 2.4× MyD88-GFP, *n* = 14,125; 10× MyD88-GFP, *n* = 1,439 versus 3.1× MyD88-GFP, *n* = 16,834). -ve, negative; +ve, positive; MW, molecular weight.

**Figure 4.**
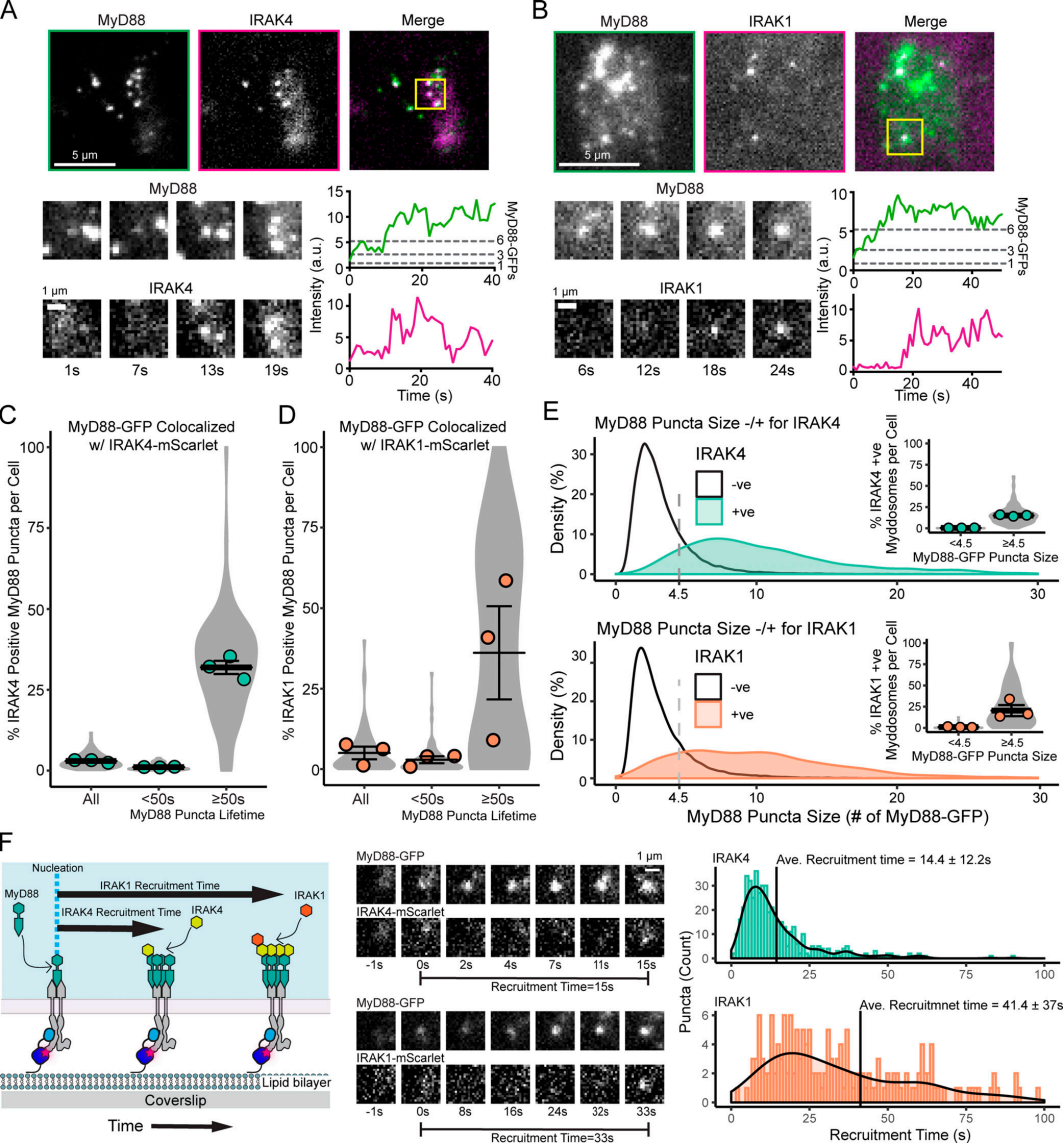
**IRAK4 and IRAK1 are recruited to larger MyD88 oligomers. (A and B)** Top: TIRF images of MyD88-GFP and IRAK4-mScarlet (A) or IRAK1-mScarlet (B). Region of interest (yellow box, merge image) shows an example of a MyD88-GFP spot colocalized with IRAK4-mScarlet (A) or IRAK1-mScarlet (B). Bottom: Time-series TIRF images from the region of interest (left) and fluorescence intensity time series (right) of MyD88-GFP and IRAK4-mScarlet (A) or IRAK1-mScarlet (B). **(C and D)** Quantification of the percentage of MyD88-GFP puncta per cell that colocalize with IRAK4 (C) or IRAK1 (D) for all puncta and puncta with lifetimes <50 s or ≥50 s. Violin plots show the distribution of individual cell measurements. Colored dots superimposed on violin plots correspond to the average value in the independent experiments (*n* = 3 for IRAK4/1; each replicate encompasses measurements from 16–34 cells). Bars represent mean ± SEM. **(E)** Density plot showing the distribution of MyD88 oligomer size (number of MyD88-GFP monomers is derived from the maximum intensity divided by the average intensity of GFP) for MyD88 puncta that are positive (+ve) or negative (-ve) for IRAK4 (top) or IRAK1 (bottom). Inset: Percentage of MyD88-GFP puncta per cell that colocalize with IRAK4 or IRAK1 with a maximum intensity of <4.5× GFP or ≥4.5× GFP. Violin plots show the distribution of individual cell measurements. Colored dots superimposed on the violin plots correspond to the mean value for the independent experiments (*n* = 3, IRAK4; *n* = 3, IRAK1). Bars represent mean ± SEM. **(F) **Analysis of IRAK4 and IRAK1 recruitment time during Myddosome assembly. Recruitment time was defined as the time interval from Myddosome nucleation (e.g., time = 0 s when MyD88-GFP puncta appears) to the appearance of IRAK4/1-mScarlet. Middle: Time series of TIRF images showing MyD88-GFP nucleation followed by IRAK4-mScarlet (top time series) or IRAK1-mScarlet (bottom time series) recruitment. Histogram of IRAK4 (*n* = 482 recruitment events, combined from 30 cells) and IRAK1 (*n* = 170 recruitment events, combined from 40 cells) recruitment times overlaid with the density plot of the distribution. Black horizontal lines on the histograms denote the average recruitment time (mean ± SD). Ave., average.

We asked whether the size of the MyD88 oligomer regulated the recruitment of downstream effectors IRAK4 and IRAK1 to the cell surface. We used CRISPR/Cas9 to engineer monoclonal EL4 cell lines to express MyD88-GFP and either IRAK4 or IRAK1 fused to the red fluorescent protein mScarlet-I in their endogenous genetic loci (Fig. S4, A–C, Fig. S7, and Fig. S8). We found that engineered EL4 cell lines expressing MyD88-GFP and IRAK4-mScarlet or IRAK1-mScarlet retained NF-κB and p38 MAPK activation as well as cytokine production after IL1 stimulation (Fig. S2, A–E). We analyzed the temporal dynamics of MyD88 and IRAK4 using multi-color TIRF microscopy in EL4 cells stimulated with our IL1β-functionalized SLB system. IRAK4-mScarlet, like MyD88, had a punctate localization pattern at the cell surface and colocalized with MyD88 (Fig. 4 A and Video 3). However, we observed that only a subset of MyD88 spots localized with IRAK4 at the plasma membrane, and MyD88 puncta appeared at the cell surface before the recruitment of IRAK4 (see example time series, Fig. 4 A; and Video 3).

**Video 3.** **IRAK4 recruitment to clusters of MyD88 (related to ****Fig. 4****).** This video shows an EL4 cell expressing MyD88-GFP (left, green channel in merge) and IRAK4-mScarlet (middle, magenta channel in merge) interacting with an IL1β-functionalized supported lipid membrane. The video illustrates the nucleation of MyD88-GFP puncta and the recruitment of IRAK4-mScarlet. Same cell as shown in Fig. 4 A. Scale bar, 5 µm.

We analyzed the temporal dynamics of MyD88 and IRAK1. Similar to IRAK4, IRAK1 had a punctate pattern at the cell surface. Only a subset of MyD88 spots localized with IRAK1 (see Fig. 4 B and Video 4). Time series analysis revealed that IRAK1 puncta appeared at the cell surface after the formation of the MyD88 puncta (Fig. 4 B). From these data, we concluded that MyD88 is recruited before IRAK4/1, and only a subset of MyD88 assemblies recruits IRAK4/1.

**Video 4.** **IRAK1 recruitment to clusters of MyD88 (related to ****Fig. 4****).** This video shows an EL4 cell expressing MyD88-GFP (left, green channel in merge) and IRAK1-mScarlet (middle, magenta channel in merge) interacting with an IL1β-functionalized supported lipid membrane. The video illustrates the nucleation of MyD88-GFP puncta and the recruitment of IRAK1-mScarlet. Same cell as shown in Fig. 4 B. Scale bar, 5 µm.

To determine which properties of MyD88 assemblies trigger IRAK4 recruitment, we quantified the percentage of MyD88-GFP puncta that colocalized with a punctum of IRAK4-mScarlet. We found that 3% (Figs. 4 C and S4 D) of MyD88 puncta per cell colocalized with IRAK4. To assess whether longer-lived MyD88 assemblies were more likely to recruit IRAK4, we compared the IRAK4 recruitment to MyD88 puncta with lifetimes of <50 s or ≥50 s. Only 1% of MyD88 puncta per cell with a lifetime of <50 s recruited IRAK4-mScarlet. In stark contrast, 32% of MyD88 puncta per cell with a lifetime of ≥50 s recruited IRAK4-mScarlet (Fig. 4 C).

We repeated this analysis using IRAK1-mScarlet. We found that 5% of MyD88-GFP puncta colocalized with a punctum of IRAK1-mScarlet, but 36% of MyD88-GFP puncta with a lifetime of ≥50 s colocalized with IRAK1-mScarlet (Figs. 4 D and S4 F). Only 3% of MyD88-GFP puncta with a lifetime of <50 s colocalized with IRAK1-mScarlet. Thus, the long-lived MyD88-GFP assemblies more efficiently recruit IRAK4 and IRAK1 to the cell surface.

We analyzed the maximum intensity of MyD88-GFP particles that colocalized with IRAK4/1. We found that IRAK4-positive MyD88 particles were brighter than IRAK4-negative MyD88 particles (Figs. 4 E and S4 E). When we normalized the MyD88 intensity to GFP, we estimated that IRAK4 was recruited to MyD88 oligomers that had an average size of 11.0× MyD88-GFP. In comparison, MyD88 particles negative for IRAK4 had an average intensity of 3.2× MyD88-GFP (Figs. 4 E and S4 E). We repeated this analysis with IRAK1 and found that IRAK1-mScarlet–positive MyD88 puncta were brighter than IRAK1-mScarlet–negative MyD88 particles (Figs. 4 E and S4 G). IRAK1-positive MyD88-GFP puncta had an average size of 12× MyD88-GFP, whereas IRAK1-negative-MyD88 puncta had an average size of 3.7× MyD88-GFP (Figs. 4 E and S4 G). Consistent with this analysis, a greater proportion of larger MyD88 oligomers colocalized with IRAK4 and IRAK1 (inset, Fig. 4 E).

### The Myddosome forms by the sequential recruitment of IRAK4 and IRAK1

We analyzed the recruitment kinetics of IRAK4 and IRAK1 to individual MyD88-GFP oligomers. We measured the time from MyD88 nucleation to the appearance of IRAK4 or IRAK1 (defined as the recruitment time; see schematic, Fig. 4 F). IRAK4 and IRAK1 recruitment time distributions had a rise and fall shape, suggesting the assembly of MyD88 was a rate-limiting step requisite for the recruitment of IRAK4 and IRAK1. IRAK4 had a distribution that peaked at ∼8 s, and an average recruitment time of 14.4 ± 12.3 s (Fig. 4 F, mean ± SD). In contrast, IRAK1 had a distribution with a peak at ∼18 s and an average recruitment time of 41.3 ± 37.5 s (Fig. 4 F, mean ± SD). The broad IRAK1 recruitment time distribution possibly reflects that, in addition to MyD88 assembly, IRAK4 assembly serves as a second rate-limiting step.

### Myddosomes are highly stable molecular assemblies that do not exchange with uncomplexed MyD88, IRAK4, or IRAK1

**Figure 5.**
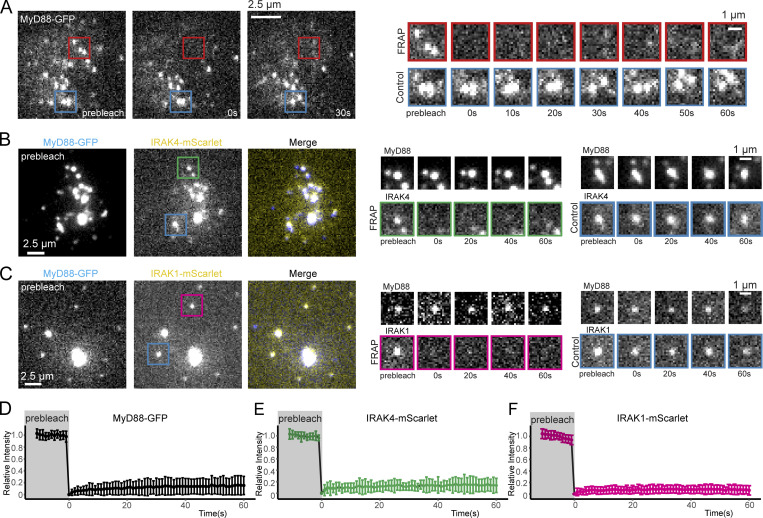
**Myddosomes are highly stable molecular assemblies that do not exchange with uncomplexed MyD88, IRAK4, or IRAK1. (A) **TIRF images of MyD88-GFP before and after (0 and 30 s) photobleaching. The red box indicates the position of the Myddosomes on which the FRAP beam was focused. The blue box indicates unbleached control. Right: Cropped time series of the photobleached and control Myddosomes. **(B and C) **TIRF images of MyD88-GFP/IRAK4-mScarlet– (B) and MyD88-GFP/IRAK4-mScarlet–(C) expressing cells before photobleaching. The green and magenta boxes indicates the position of the IRAK4-mScarlet– and IRAK1-mScarlet–labeled Myddosome on which the FRAP beam was focused, respectively. The blue box indicates unbleached control. Right: Cropped time series of the photobleached and control IRAK4-mScarlet– (B) or IRAK1-mScarlet– (C) labeled Myddosome. **(D–F)** Normalized recovery curves for MyD88 (*n* = 61 cells), IRAK4 (*n* = 22 cells), and IRAK1 (*n* = 53 cells). All yielded a maximal recovery of <20% during the 60 s of observation.

We applied fluorescent recovery after photobleaching to analyze the internal molecular dynamics of assembled Myddosomes. EL4 cells endogenously expressing MyD88-GFP were incubated with IL1β-functionalized SLBs for 15 min to allow Myddosomes to form. We selected and photobleached large MyD88-GFP puncta. The photobleached MyD88 puncta did not recover over the experimental time course (60 s after bleaching; Fig. 5 A and Video 5). We applied the same analysis to EL4 cells endogenously expressing IRAK4-mScarlet and IRAK1-mScarlet. Similar to MyD88-GFP, IRAK4 and IRAK1 puncta did not recover after photobleaching (Fig. 5, B and C). Our FRAP analysis of multiple Myddosomes revealed that the core components of MyD88, IRAK4, and IRAK4 do not undergo dynamic exchange (Fig. 5, D–F).

**Video 5.** **FRAP analysis of MyD88-GFP (related to ****Fig. 5****).** This video shows an EL4 cell expressing MyD88-GFP in which a region of interest (red box) is photobleached. The video illustrates that Myddosomes show no fluorescence intensity recovery after photobleaching. Same cell as shown in Fig. 5 A. Scale bar, 5 µm.

### IRAK4 knockout (KO) leads to super MyD88 oligomers

**Figure S5.**
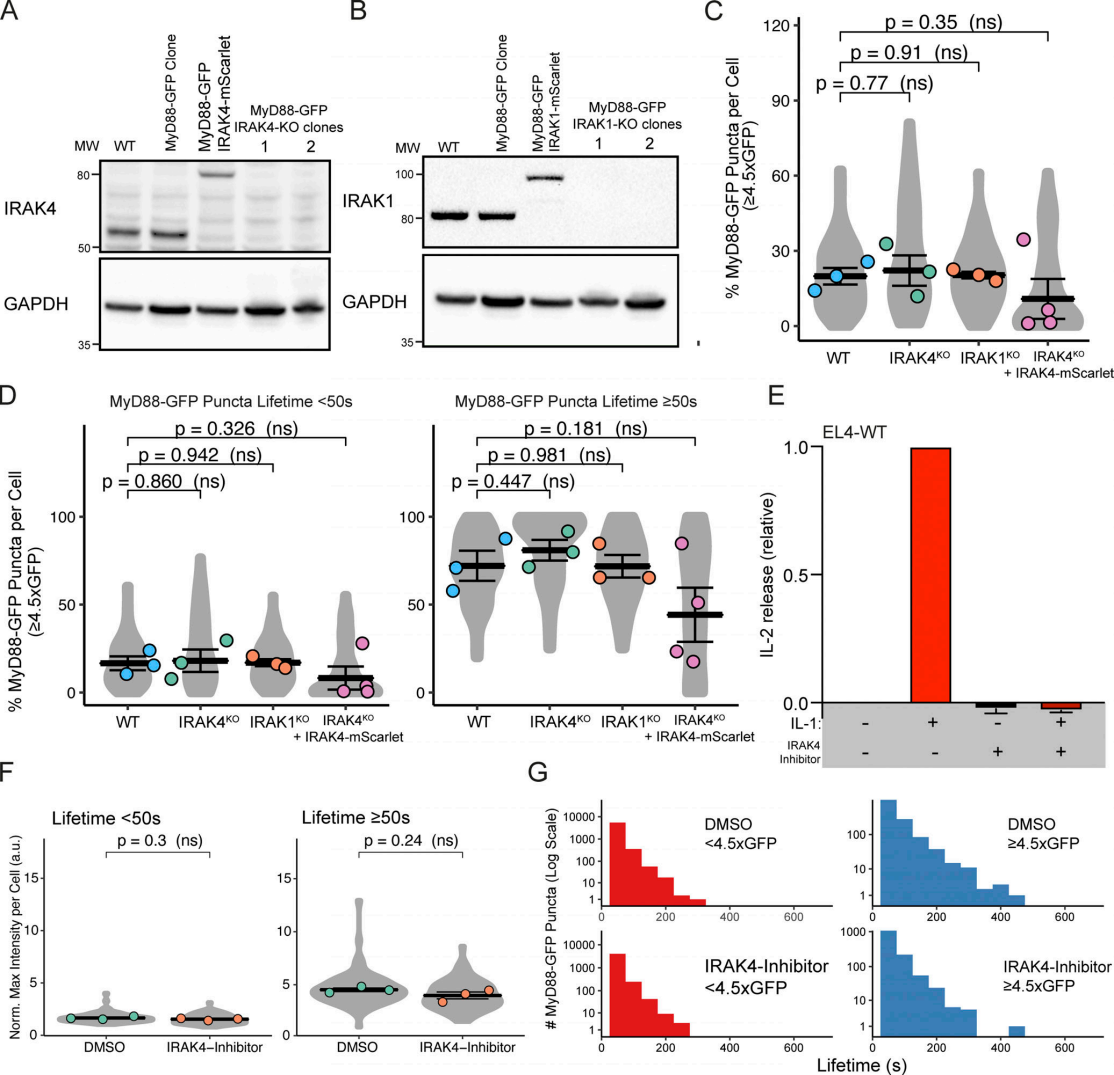
**CRISPR/Cas9 KO IRAK4 and IRAK1 EL4 cell lines, and MyD88-GFP dynamics in KO cell lines. (A and B)** Validation of IRAK4 and IRAK1 KO cell lines. Western blot analysis of two monoclonal EL4 IRAK4 KO (A) and IRAK1 KO (B) cell lines. **(C)** The percentage per cell of large MyD88 oligomers in WT, IRAK4 KO, and IRAK1 KO EL4 cell lines. Quantification of the percentage (%) per cell of MyD88-GFP puncta with a maximum intensity ≥4.5× GFP. Violin plots show the distribution of the cell data. Data points superimposed on violin plots are the averages of independent experimental replicates. Bars represent mean ± SEM (*n* = 3 or 4 experimental replicates, with >10 cells measured per replicate). **(D)** The percentage per cell of large MyD88 oligomers is equivalent for short- and long-lived MyD88-GFP puncta across WT and KO cell lines. Quantification of the proportion (%) per cell of MyD88-GFP puncta with a maximum intensity of ≥4.5× GFP categorized by lifetimes of <50 s or ≥50 s. Violin plots show the distribution of the cell data. Data points superimposed on the violin plots are the averages from independent experiments. Bars represent mean ± SEM (*n* = 3 or 4 experimental replicates, with >10 cells measured per replicates). WT and KO image means were compared using an unpaired Student's *t* test. **(E)** IL2 release in EL4-WT cells treated with IRAK4 inhibitor. EL4 cells were pretreated with DMSO or IRAK4 inhibitor (20 µm) for 30 min. Cells were then left untreated or stimulated with 10 ng/ml of IL1 for a further 24 h. IL2 release was assayed by ELISA. Values for IRAK4 inhibitor cells shown relative to DMSO-only treated cells. Average values calculated from three independent experiments. Bars represent mean ± SEM. **(F)** Quantification of the maximum intensity of MyD88-GFP puncta per cell with lifetimes of <50 s or ≥50 s. Violin plots show the distribution of individual cell measurements. Colored dots superimposed on violin plots correspond to the average value in the independent experiments (*n* = 3 experimental replicates; encompasses measurements from 19–39 cells). Bars represent mean ± SEM. P values were calculated using a two-tailed unpaired Student’s *t* test. **(G)** Distribution of lifetimes for tracked MyD88-GFP puncta with a maximum intensity of <4.5× or ≥4.5× GFP in DMSO control (*n* = 91,876 for <4.5× GFP and *n* = 4,836 for ≥4.5× GFP), and IRAK4 inhibitor (*n* = 64,822 for <4.5× GFP and *n* = 3,828 for ≥4.5× GFP) –treated cells. MyD88-GFP puncta lifetimes collated from three experimental replicates. Max, maximum; Norm., normalized; MW, molecular weight.

**Figure 6.**
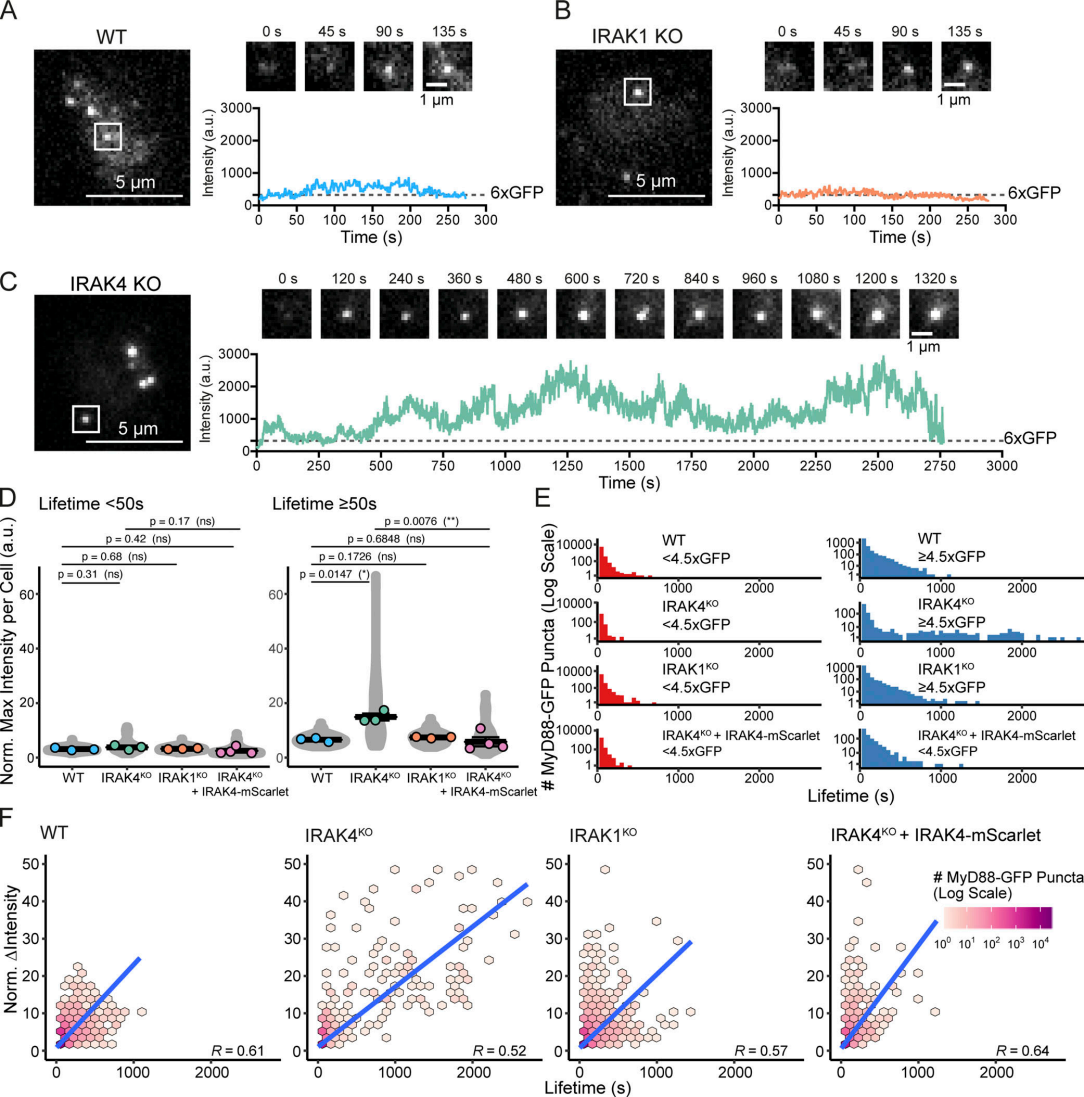
**IRAK4 KO leads to super MyD88 oligomers. (A–C)** TIRF images of MyD88-GFP in EL4 WT (A), IRAK1 KO (B), and IRAK4 KO (C) cells. Time-series TIRF images from the region of interest (white box) showing representative MyD88 puncta. A fluorescence intensity time trace from each time series is shown below. **(D)** Quantification of the maximum intensity of MyD88-GFP puncta per cell with lifetimes of <50 s or ≥50 s. Violin plots show the distribution of individual cell measurements. Colored dots superimposed on violin plots correspond to the average value in the independent experiments (*n* = 3 or 4 experimental replicates; encompasses measurements from 10 to 47 cells). Bars represent mean ± SEM. P values were calculated using a two-tailed unpaired Student's *t* test. **(E)** Distribution of lifetimes for tracked MyD88-GFP puncta with a maximum intensity of <4.5× or ≥4.5× GFP in WT (*n* = 73,180 for <4.5× GFP and *n* = 24,631 for ≥4.5× GFP), IRAK4 KO (*n* = 18,478 for <4.5× GFP and *n* = 9,795 for ≥4.5× GFP), and IRAK1 KO (*n* = 63,382 for <4.5× GFP and *n* = 19,735 for ≥4.5× GFP) and IRAK4 KO + IRAK4-mScarlet (*n* = 18,764 for <4.5× GFP and *n* = 5,627 for ≥4.5× GFP) cells. MyD88-GFP puncta lifetimes collated from 3 or 4 experimental replicates. **(F)** 2D histogram of MyD88-GFP puncta lifetime versus change in fluorescent intensity for WT (*n* = 64,149), IRAK4 KO (*n* = 13,960), IRAK1 KO (*n* = 54,551), and IRAK4 KO + IRAK4-mScarlet (*n* = 18,154) cells. Linear fit is shown as a blue line. The coefficient used is Spearman’s rank correlation coefficient. Max, maximum; Norm., normalized.

We used CRISPR/Cas9 to generate IRAK4/1 KO cell lines (Fig. S5, A and B; and Fig. S9). In agreement with previous studies (DeFelice et al., 2019; Suzuki et al., 2002), we found that IRAK4 and IRAK1 KO EL4 cells could not activate NF-κB or p38 MAPK signaling or release IL2 when stimulated with IL1β (Fig. S2, A–E). We assayed the temporal dynamics of MyD88-GFP puncta in the IRAK4 and IRAK1 KO cell lines. We observed that IL1β stimulation induced the formation of MyD88-GFP puncta in IRAK4 and IRAK1 KO cell lines (Fig. 6, A–C; and Video 6). Thus, the loss of IRAK4 and IRAK1 does not inhibit the recruitment and oligomerization of MyD88 at activated IL1Rs. However, we did observe that IRAK4 KO cells formed larger (e.g., brighter) MyD88-GFP assemblies. The intensity of MyD88 puncta in IRAK4 KO cells suggests they contain a greater stoichiometry of MyD88 than the 6–8 MyD88s found in purified Myddosome complexes (Lin et al., 2010; Fig. 6, A–C).

**Video 6.** **MyD88-GFP dynamics in WT, IRAK4 KO, and IRAK1 KO EL4 cells (related to ****Fig. 6****).** This video shows MyD88-GFP in WT (left), IRAK1 KO (middle), and IRAK4 KO (right) EL4 cells landing on an IL1β-functionalized supported lipid membranes. White box in each panel indicates example cells shown in Fig. 6 A. Scale bar, 5 µm.

**Figure S6.**
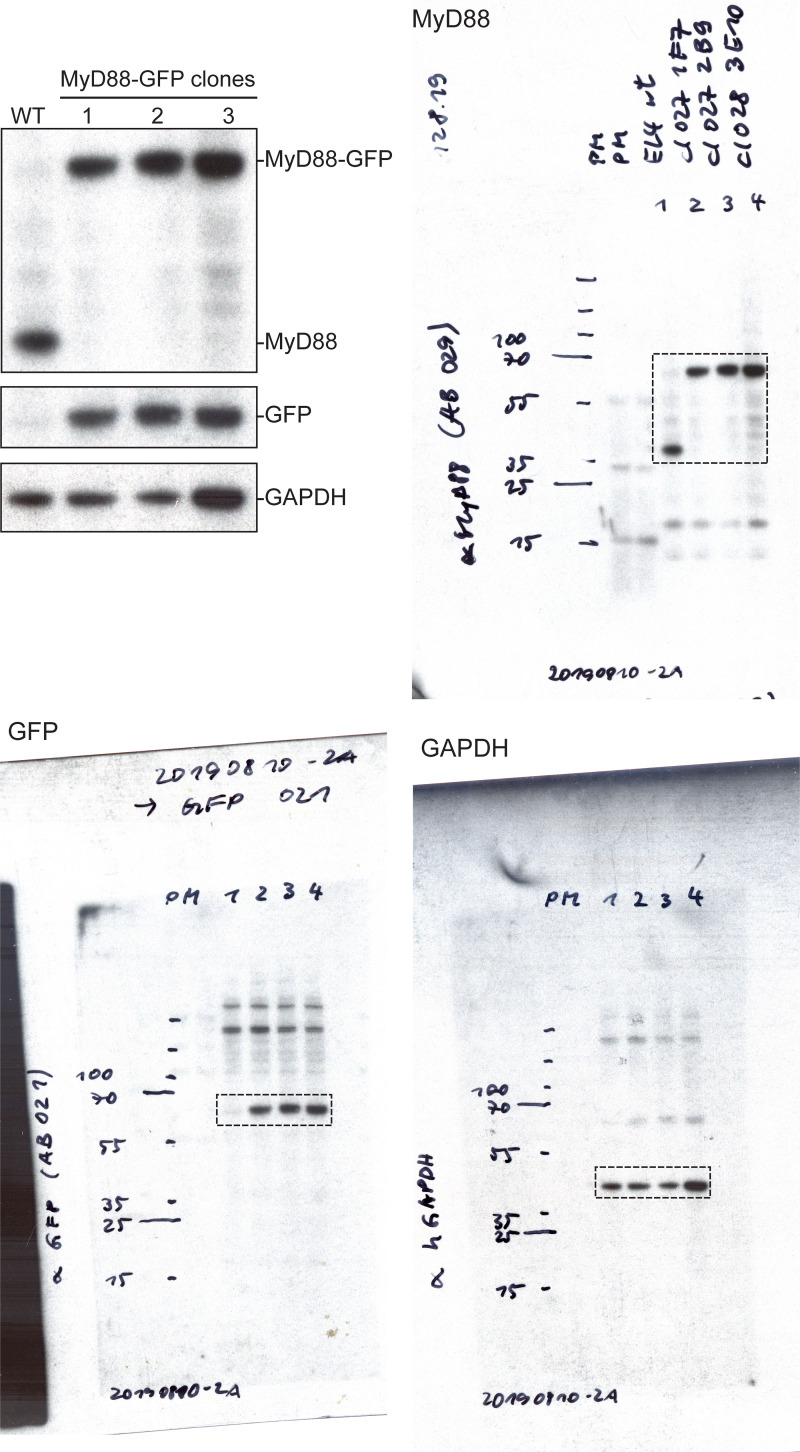
**Full-length Western blots from ****Fig. S1 D****.**

**Figure S7.**
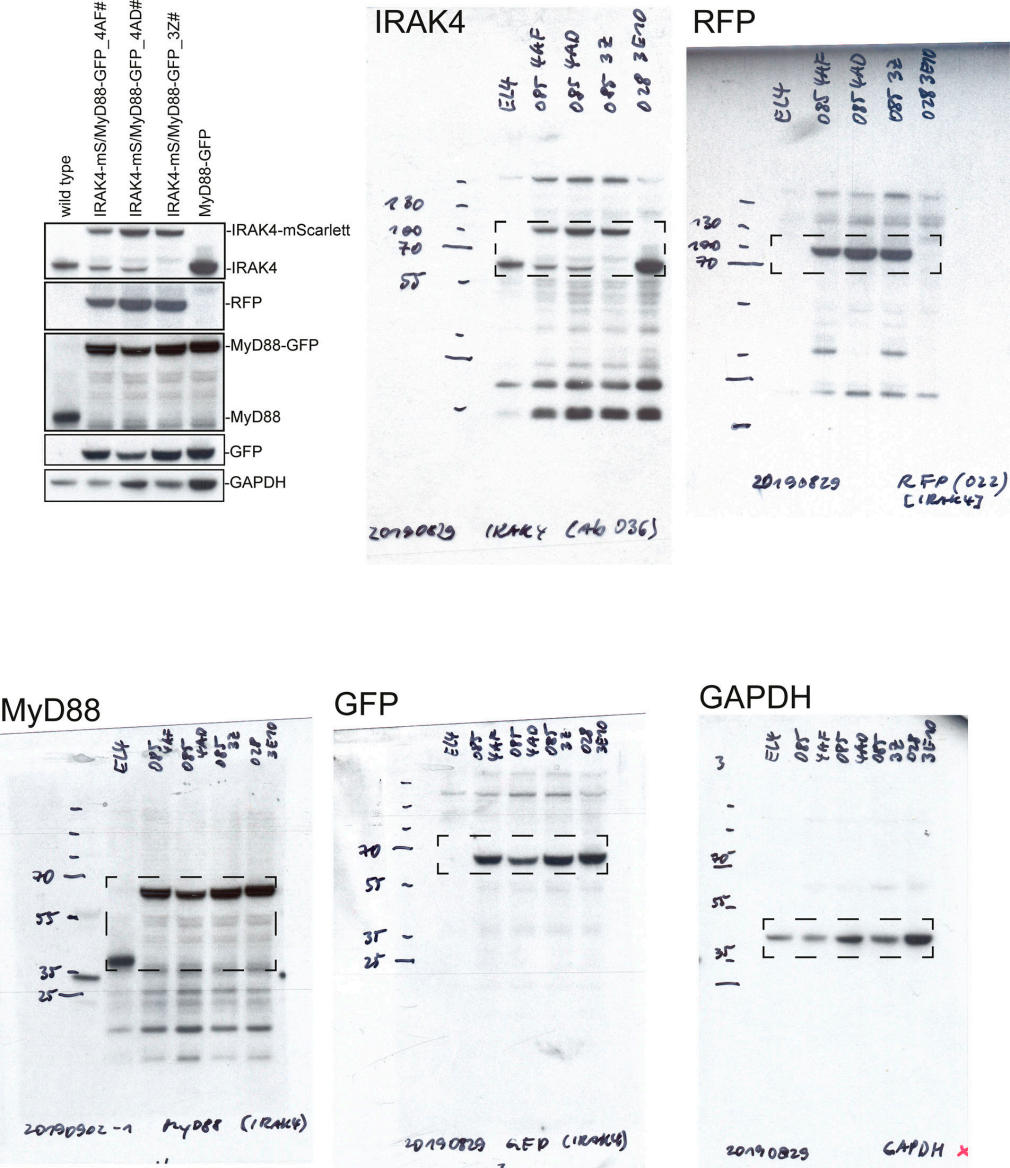
**Full-length Western blots from ****Fig. S4 B****.**

**Figure S8.**
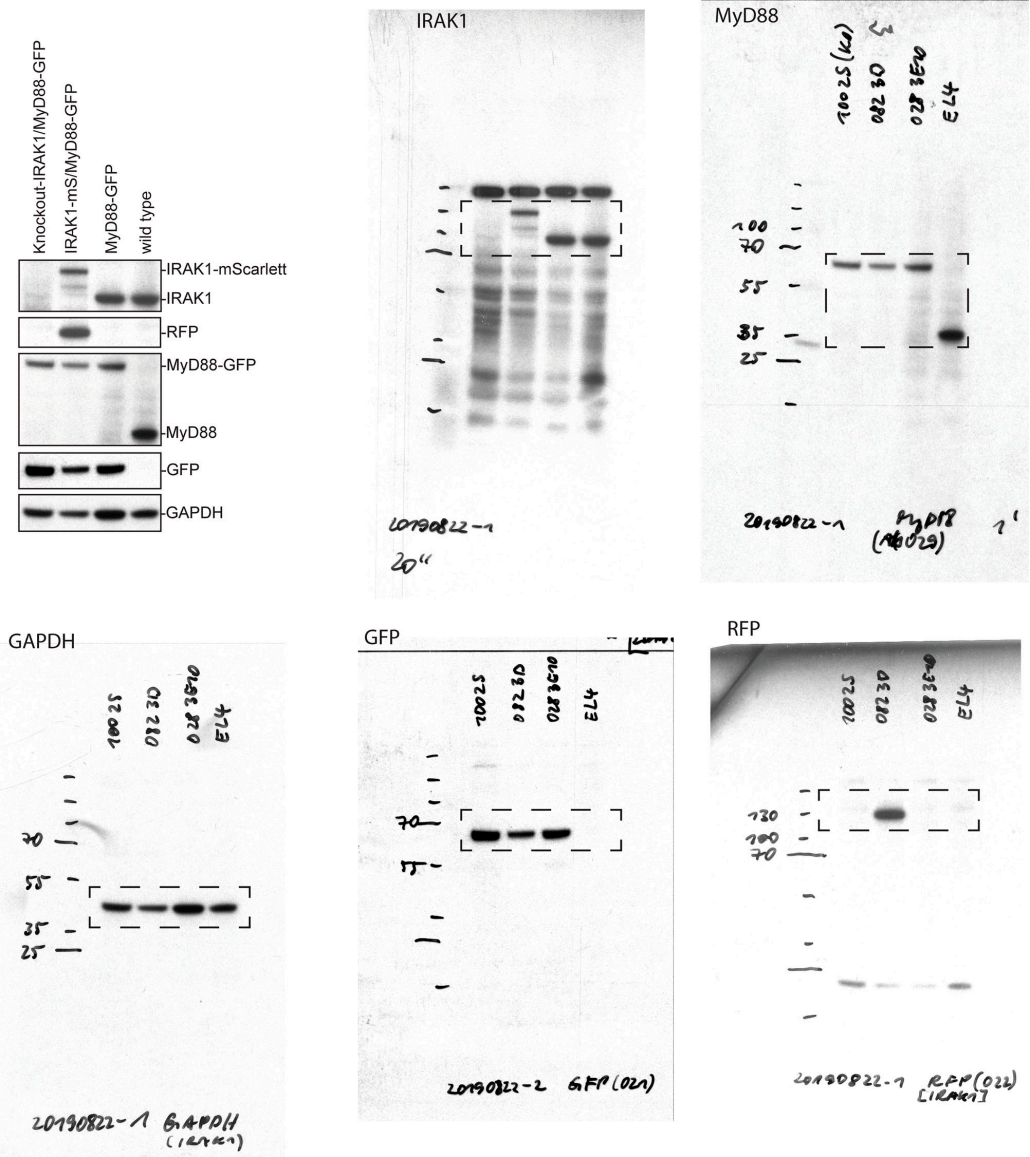
**Full-length Western blots from ****Fig. S4 C****.** Note Western blot images have been reflected around its vertical axis for Fig. S4 C to enhance clarity.

**Figure S9.**
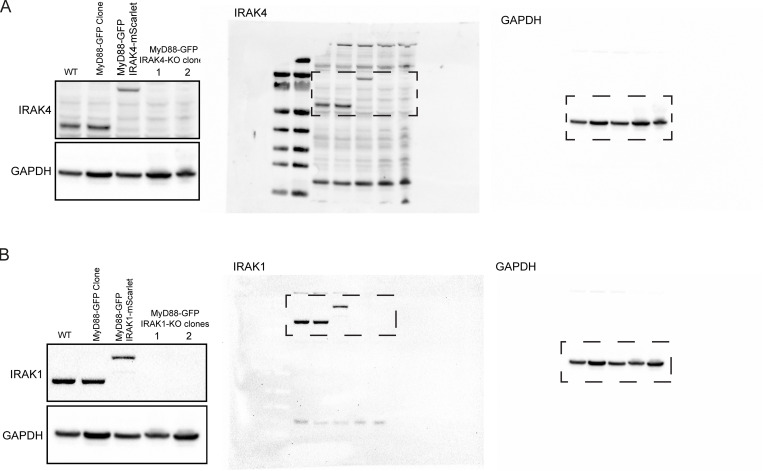
**Full-length Western blots from ****Fig. S5, A and B****, for IRAK4 and IRAK1 KO cells.**

We analyzed the distribution of MyD88 oligomer sizes in IRAK4 and IRAK1 KO cell lines. The proportion of larger MyD88 oligomers (i.e., ≥4.5× GFP; Fig. 3 B) per cell in IRAK4 and IRAK1 KO cell lines was equivalent to that of WT EL4 cells (Fig. S5, C and D). The size of MyD88-GFP puncta with lifetimes of <50 s was equivalent, with no statistically significant difference between WT and KO cells (MyD88-GFP sizes were 3.12× for WT, 3.78× for IRAK4 KO, and 3.26× for IRAK1 KO; Fig. 6 D). In contrast, MyD88 puncta with lifetimes of ≥50 s were twice as large in IRAK4 KO cells compared with WT and IRAK1 KO cells (MyD88-GPF sizes were 6.58× for WT, 14.9× for IRAK4 KO, and 7.40× for IRAK1 KO; Fig. 6 D). Smaller MyD88-GFP multimers had a similar lifetime distribution across WT and IRAK4 and IRAK1 KO cell lines. However, MyD88-GFP puncta ≥4.5× GFP had extended lifetimes in the IRAK4 KO cells, with several that could be tracked for >30 min (Fig. 6 E).

We reconstituted IRAK4 KO cells with IRAK4-mScarlet expressed from a lentiviral vector. IRAK4 KO cells transduced with IRAK4-mScarlet formed MyD88-GFP puncta that had no statistical difference in size from WT cells (Fig. 6 D). IRAK4-mScarlet expression decreased the lifetime MyD88-GFP puncta ≥4.5× GFP in IRAK4 KO cells (Fig. 6 E). We tested whether pharmacological inhibition of IRAK4 kinase activity can phenocopy the effect of IRAK4 KO on MyD88 oligomerization. We incubated MyD88-GFP EL4 cells with IRAK4 kinase inhibitor (PF-06650833 at 20 µM; Lee et al., 2017; a concentration that inhibited IL2 release; Fig. S5 E) for 30 min before assaying MyD88 dynamics in the presence of the inhibitor. We found no difference in MyD88 oligomer size and lifetimes between cells incubated with DMSO or the IRAK4 inhibitor (Fig. S5, F and G). Therefore, IRAK4-mScarlet expression can rescue the effects on MyD88-GFP oligomer size and lifetime in IRAK4 KO cells, and pharmacological inhibition of IRAK4 kinase activity does not induce the formation of super MyD88 oligomers.

We examined the correlation between growth in intensity and lifetime. Similar to our previous analysis (Fig. 3 F), we measured a positive correlation between an increase in intensity and the lifetime for MyD88-GFP tracks for WT and the IRAK4 and IRAK1 KO cell lines (R ≥ 0.55 across all WT and KO cell lines; Fig. 6 F). However, in IRAK4 KO cells, we observed that a portion (*n* = 110 out of *n* = 28,273 total MyD88-GFP puncta) of very long-lived events (1,000 s) had larger changes in intensity (>20× GFP) that were not observed in WT cells (Fig. 6 F). We did not find MyD88-GFP puncta with these characteristics in IRAK4 KO cells reconstituted with IRAK4-mScarlet (Fig. 6 F). This analysis revealed that the greater lifetimes of MyD88-GFP puncta in the IRAK4 KO background correlated with increased growth in intensity (R = 0.55).

**Figure 7.**
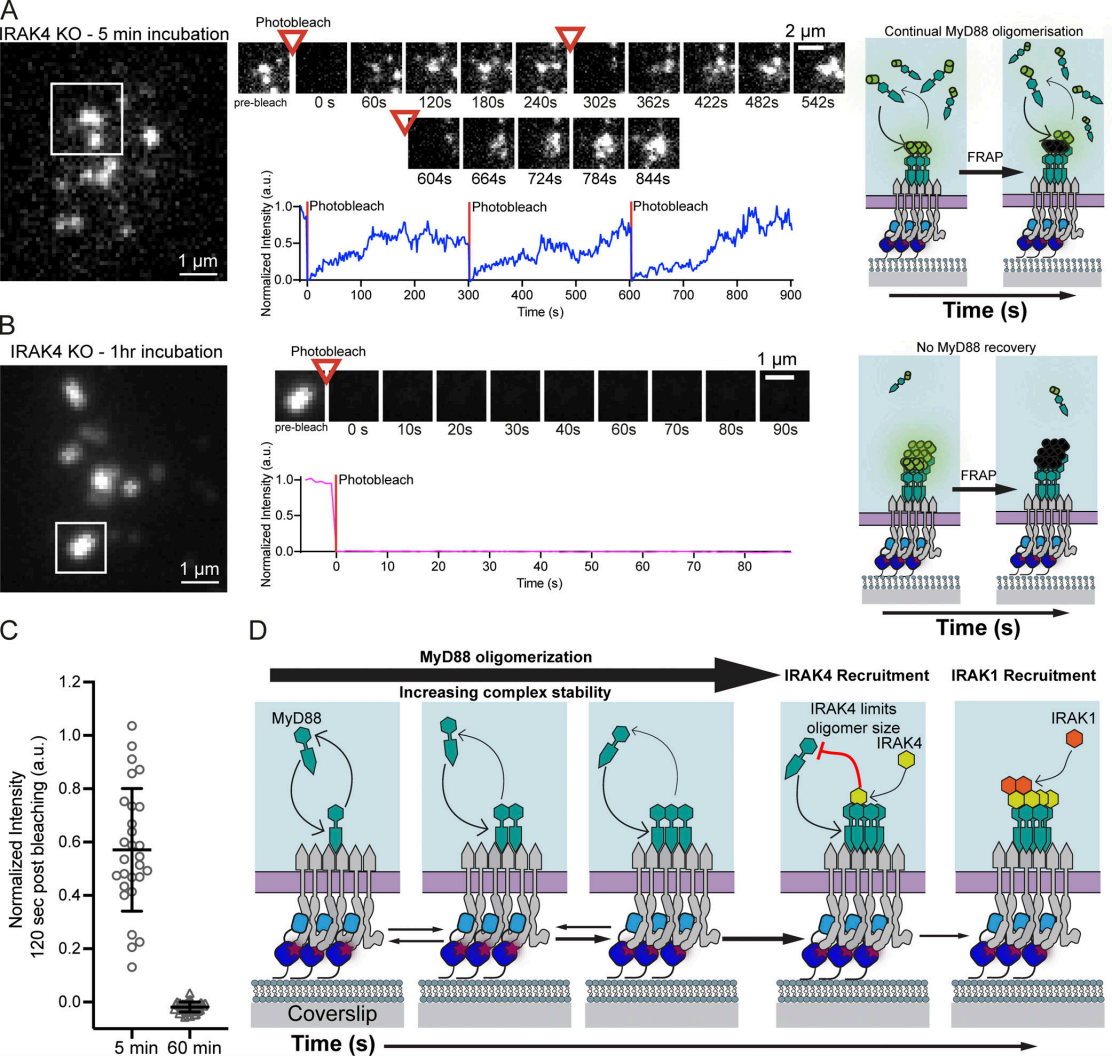
**Recovery after photobleaching of super MyD88 oligomers in IRAK4 KO cells is time-dependent. (A and B)** TIRF images of MyD88-GFP in IRAK4 KO cells after 5 min (A) or 1 h incubation on IL1 SLBs. The white boxes indicate the position of photobleached Myddosomes. Middle: Cropped time series of the photobleached Myddosomes. Right: Schematics show possible explanation of fluorescence recovery due to continual MyD88 oligomerization at early time points (A). Conversely, MyD88 depletion after 1 h could explain loss of fluorescence recovery (B). **(C)** Quantification of super MyD88 oligomers recovery 2 min after photobleaching after 5 min or 1 h incubation. Bar represents mean ± SD; scatter plot symbols represent single MyD88-GFP oligomer photobleaching recovery measurements (*n* = 28 and *n* = 42 MyD88-GFP puncta for 5 min and 60 min, respectively). **(D)** Speculative model describing Myddosome assembly. MyD88 recruitment to the IL1-bound IL1R nucleates MyD88 oligomerization. Initially, the small oligomer of MyD88 is unstable and can disassemble. However, as MyD88 oligomer size increases, so does complex stability, and the formation of larger MyD88 complexes triggers downstream signaling in the form of the sequential recruitment of IRAK4 followed by IRAK1.

We next performed FRAP on MyD88 super-assemblies in the IRAK4 KO cells. We photobleached MyD88 super-assemblies that formed within 5 min of cells landing on the bilayer (Fig. 7 A). In contrast with assembled Myddosomes in WT cells, these large MyD88 assemblies recovered fluorescence, and in some instances could be photobleached multiple times. In contrast, after 1 h of stimulation, we found MyD88 super-assemblies did not recover after photobleaching (Fig. 7 B). MyD88 super-assemblies recovered fluorescence to 57 ± 23% of the prebleach intensity after 2 min (mean ± SD). In contrast, after 1 h, these super-assemblies did not recover intensity (−1.9 ± 1.9% of the prebleach intensity at 2 min after photobleaching, mean ± SD; Fig. 7 C).

In conclusion, MyD88 polymerization was unregulated and led to the formation of super-MyD88 oligomers in IRAK4 KO cells. These MyD88 super-assemblies showed a time-dependent fluorescence recovery: initially super-assemblies recovered fluorescence after photobleaching, but after an hour of incubation, photobleaching fluorescence recovery was negligible. This suggests that MyD88 oligomer growth, even in the IRAK4 KO cells, was finite. Taken together, we argue that IRAK4 regulates MyD88 oligomer size.

## Discussion

The discovery of innate immune receptors and SMOCs argued that macromolecular assembly, in addition to enzymatic activity, can transduce intracellular signaling. However, how does the dynamic process of oligomerization transmit a biochemical signal? To address this question, we developed a live-cell assay to investigate the assembly kinetics of Myddosome SMOCs in live cells stimulated with IL1β. We found that Myddosome assembly in IL1R signaling is dependent on the formation of MyD88 oligomers of a critical size. The formation of large MyD88 oligomers functions as a biochemical threshold that is overcome to activate downstream signaling effectors IRAK4 and IRAK1. Interestingly, MyD88 oligomer size is sensed and controlled by IRAK4 (Fig. 7 D). Given that multiple innate immune receptors use SMOCs, similar mechanistic principles might operate in other innate immune signaling pathways.

Here we show that small MyD88 oligomers are kinetically unstable and had short lifetimes at activated IL1Rs (Figs. 2 C and 3 D). This instability potentially serves as a safety switch that prevents MyD88 signaling in the absence of stimuli. Relatively few nucleated MyD88 oligomers transitioned to larger signaling-competent oligomers (Fig. 3 C). The low probability of small oligomers transitioning to a large signaling-competent MyD88 oligomers creates a time delay between IL1R activation and signal transduction. This could ensure cells only activate from sustained IL1R activation resulting from a persistent microbial or environmental threat. This might prevent harmful physiological consequences of auto-activation. Notably, some oncogenic MyD88 mutations have an increased propensity to oligomerize (O’Carroll et al., 2018). These mutations result in sustained NF-κB signaling in the absence of IL1R stimulation and are driver mutations in certain B cell lymphomas (Ngo et al., 2011).

We argue that the necessity to suppress auto-activation and be reactive to IL1R activation constrains MyD88 self-assembly. Consistent with this argument, IL1R activation induces the recruitment and self-assembly of MyD88 at the plasma membrane (Fig. 2 C). MyD88 oligomerization is initially reversible, and small oligomers (<4 MyD88s) are kinetically unstable (Fig. 3, A and D; and Fig. 7 D). Smaller MyD88 oligomers failed to recruit IRAK1 and IRAK4, suggesting limited signaling output (Fig. 4 E), and not every interaction between MyD88 and IL1R leads to productive signaling.

We found that larger MyD88 oligomers consisting of >4 MyD88s had increased lifetimes (Fig. 3 D), and were more likely to coassemble with IRAK4 and IRAK1 (Fig. 4, C–E). In this manner, MyD88 oligomer size could act as a physical threshold that must be reached to activate downstream IL1R signaling. The assembly of larger stable MyD88 oligomers also correlated with p38 MAPK signaling (Fig. 3, G and H). Therefore, we argue that the formation of larger, stable MyD88 oligomers is a decision-making step in IL1R signal transduction.

Many SMOCs are composed of DD-containing proteins, and structural studies have revealed these effectors can form helical oligomeric complexes (Ferrao and Wu, 2012). Like IL1Rs and MyD88, many innate immune receptors and their binding effectors do not contain enzymatic activity, but coassemble and activate downstream enzymes. Our data, coupled with structural studies, suggest a general mechanism where DD polymer size can create thresholds for triggering the next step of a signaling pathway (Wu, 2013). For example, small oligomers of AIM2 or NLRP3 receptors cap oligomers of the ASC signaling adaptor (Lu et al., 2014). This suggests that AIM2/NLRP3 receptors oligomers assembled first and that reaching a requisite size triggers ASC assembly. The TNF receptor signaling adaptor FADD forms an oligomeric complex at the base of caspase-8 filaments. This suggests a required FADD oligomer size is necessary to nucleate caspase-8 assembly and activation (Fu et al., 2016).

Consistent with previous studies on TLR signaling (Bonham et al., 2014), we have shown that assembled Myddosomes are only detected after IL1 stimulation. Using live-cell image analysis, we measured the kinetics and precise molecular choreography of Myddosome assembly as it pertains to IL1R signaling. We directly visualized the sequential assembly of MyD88, followed by IRAK4, and then IRAK1 into Myddosomes over ∼1 min time scale (Fig. 4 F). Microscopy and biochemical analysis have suggested that oligomers of MyD88 might be present in the cytosol before TLR/IL1R activation (Moncrieffe et al., 2020). While our data do not exclude the presence of preassembled MyD88 oligomers, we found MyD88 oligomerization was inducible and preceded IRAK4/1 recruitment (Fig. 3 A; and Fig. 4, A and B). From our data, we conclude that Myddosomes assemble on demand with an ordered molecular choreography after IL1R activation.

The Myddosome is a signaling complex used by nearly all members of the TLR/IL1R superfamily (Gay et al., 2014; Fitzgerald and Kagan, 2020). Whether different TLRs/IL1Rs have increased or decreased affinity to MyD88 and therefore can enhance or slow the kinetics of MyD88 oligomerization remains unknown. Kinetic differences in assembly between different TLR/IL1Rs could be attributed to differences in Myddosome composition and subcellular location of signaling. Unlike IL1Rs, many TLRs require the TIR domain sorting adaptor TIRAP to signal (Horng et al., 2002). TIRAP functions as a sorting adaptor for TLR signaling via a lipid interaction domain that can bind to a wide variety of phosphoinositides, and allows TIRAP to regulate Myddosome formation at the plasma membrane and intracellular membranes enriched in phosphoinositides (Kagan and Medzhitov, 2006; Bonham et al., 2014).

These distinctions between TLRs and IL1R make it difficult to extrapolate how the measurements reported here for IL1R relate to TLR Myddosome assembly and signaling. Whether or not TIRAP potentiates MyD88 oligomerization to overcome the kinetic bottleneck of forming stable oligomers remains unclear. Furthermore, it is possible that intracellular membranes are enriched in additional regulatory factors that change the kinetics of Myddosome assembly. Future studies are needed to determine how the kinetics of Myddosome assembly varies across the TLR/IL1R superfamily.

The induction of inflammation is a required step for the initiation of a complete immune response; therefore, the high stability of SMOCs could be a biophysical feature that ensures cells activate a full response. We found that photobleached Myddosome components do not recover fluorescence intensity (Fig. 5). These results argue that the Myddosome in IL1R signaling is a stable macromolecular structure with no measurable molecular turnover. It is unknown whether other DD higher-order assemblies have similar kinetic stability. However, DD complexes such as the Fas-FADD, FADD-caspase8, and PIDDosome have highly ordered quaternary structures (Wang et al., 2010; Park et al., 2007; Fu et al., 2016), where subunits have multiple interaction interfaces. This feature suggests a similar intrinsic stability. The prevalence of these complexes in immune signaling pathways suggests this stability might be advantageous to signal transduction. A low dissociation rate might increase the time frame in which downstream reactions, such as the activation of TRAF proteins and ubiquitin ligases (Deng et al., 2000), can be achieved.

Structural studies on DD superfamily signaling proteins have revealed complexes with defined stoichiometric ratios of effectors as well as open-ended filamentous structure. Like the Myddosome, the Fas-FADD complexes have defined ratios of 5:5 (Wang et al., 2010). However, how can this be reconciled with DD proteins, such as MyD88, and FADD forming open-ended filaments (Moncrieffe et al., 2020; Fu et al., 2016)? In this study, we observed that loss of IRAK4 results in MyD88 super-assemblies that contain a greater copy number of MyD88 than those observed in structural studies (Lin et al., 2010). These super-assemblies are a result of unchecked MyD88 oligomerization in response to IL1 stimulation (Fig. 6). FRAP analysis of these MyD88 super-assemblies revealed a time-dependent fluorescence recovery (Fig. 7, A–C). Given that we could follow single super MyD88 oligomers growing in intensity for 30 min (Fig. 6 C), this time dependence might reflect that initially fluorescence recovery is due to the addition of new MyD88 monomers to the bleached MyD88 oligomer (see schematic, Fig. 7 A). Unchecked MyD88 oligomerization in IRAK4 KO cells potentially depletes the available pool of MyD88 monomers, stalling MyD88 oligomer growth, resulting in no fluorescence recovery after 1 h incubation (Fig. 7 C). It is possible that alternate mechanisms, in addition to IRAK4, regulate MyD88 oligomerization (such as post-translational modifications) and could explain the loss of fluorescence recovery after 1 h. Nonetheless, these results, taken together, suggest that MyD88 can assemble into extended oligomers in vivo, and IRAK4 is a regulator of MyD88 oligomer size in IL1R signal transduction.

The sequential recruitment of IRAK4/1 to MyD88 puncta (Fig. 4 F) and super MyD88 assemblies in IRAK4 KO cells (Fig. 6 C) raises the possibility that IRAK4 senses MyD88 oligomer size and restricts further assembly by capping the growing end of the MyD88 filament (Fig. 7 D). In vitro studies have observed the dissolution of MyD88 DD filaments into smaller filaments when incubated with IRAK4 DD (Moncrieffe et al., 2020). One possibility is that IRAK4 regulates the size of the MyD88 oligomers via heterotypic DD interactions; however, the precise mechanism remains unknown. Inhibition of the IRAK4 kinase domain did not result in the formation of super MyD88 oligomers (Fig. S5 F), suggesting that kinase activity is not required. Whole classes of effectors regulate cytoskeletal polymer size and growth (Mohapatra et al., 2016). We speculate that effectors analogous to those in cytoskeletal systems could regulate SMOC polymer dynamics. Regulators of SMOC size and assembly could be critical to building precise signal thresholds for cellular activation.

We conclude that MyD88 oligomerization is a decision-making step in IL1R Myddosome signaling. We propose that the macromolecular assembly of proteins in itself can conceptually be considered a signal transduction step, analogous to phosphorylation in many signaling pathways. Beyond IL1R, multiple innate immune signaling pathways, such as inflammasome receptors, RIG-1 DNA sensors, and TNF receptors, have an equivalent biochemical architecture that consists of receptors and signaling adaptors that self-assemble (Kagan et al., 2014). These diverse receptor systems possibly transduce signals with a similar molecular choreography that begins with the formation of unstable small oligomers that mature into stable larger oligomers that in turn activate downstream signaling. Thus, stepwise assembly, as we have found here for the Myddosome, is likely to be a fundamental feature of SMOC signaling pathways. The study of these diverse innate immune receptors with high spatial-temporal resolution microscopy will lead to a deeper understanding of how protein oligomerization functions in signal transduction.

## Methods

### Cell culture

EL4.NOB1 WT (ECACC, and referred to as EL4 in the paper) and gene-edited cells were grown in RPMI (Thermo Fisher Scientific) with 10% FBS (Biozol) supplemented with 2 mM L-glutamine. EL4 cultures were maintained at a cell density of 0.1–0.5 × 10^6^ cells/ml in 5% CO_2_, 37°C. HEK-293T cells (American Type Culture Collection) were grown in DMEM (Thermo Fisher Scientific) supplemented with 2 mM L-glutamine and 10% FBS. All cells were determined to be negative for mycoplasma using the MycoAlert detection kit (Lonza).

### Homology-directed repair (HDR) DNA template design for CRISPR/Cas9 endogenous labeling

Plasmid DNA repair templates were designed using a pMK (Life Technologies) vector backbone. Silent mutations were included in the homology arms to remove single-guide RNA (sgRNA) target sites and avoid Cas9 cleavage of the repair template. Homology arms were amplified from EL4 Genomic DNA or ordered as gBlock from Integrated DNA Technologies, and assembled with DNA fragments encoding a fluorescent protein tag (e.g., mEGFP or mScarlet-I; Bindels et al., 2017) and pMK plasmid backbone using Gibson Assembly. All HDR template plasmids were sequence-verified. Full details of the HDR DNA template plasmid construction are given below.

### pMK-MyD88-mEGFP-HDR

5′ and 3′ homology arms were designed from the mouse *MyD88* gene (ENSMUSG00000032508) covering a distance of 1,015 bp and 1,069 bp on either side of the TGA stop codon. mEGFP was inserted between these homology arms and fused to the MyD88 C terminus via a 3×(Gly-Gly-Ser) linker. The following primers were used: 5′ homology arm, 5′-ATG​CCT​CCA​TCA​TAG​TTA​ACC​GGG​ATT​TC-3′ and 5′-GGG​CAG​GGA​CAA​AGC​CTT​GGC​AAG​GCG​GG-3′; 3′ homology arm, 5′-AGA​TGA​CAC​TGA​GAA​CCC​TAT​GTA​TGT​CAG​TCT​GTC​TGT​GTT​CTT​CCG​CT-3′ and 5′-CCT​GCA​GCT​GCT​TTG​TGG​GGC​GAA​GCC​AAA​CAG-3′; mEGFP, 5′-CCC​GCC​TTG​CCA​AGG​CTT​TGT​CCC​TGC​CCG​GAG​GAT​CTG​GTG​GAT​CAG​GTG​GAA​GTG​TGA​GCA​AGG​GCG​AGG​AGC​TGT​TCA​CCG​GG-3′ and 5′-AGG​GTT​CTC​AGT​GTC​ATC​TTC​ACT​TGT​ACA​GCT​CGT​CCA​TGC​CGA​GAG​TG-3′; and pMK vector backbone, 5′-CCC​CAC​AAA​GCA​GCT​GCA​GGC​ATG​GTC​ATA​GCT​GTT​TCC-3′ and 5′-CCG​GTT​AAC​TAT​GAT​GGA​GGC​ATC​TGG​CCG​TCG​TTT​TAC​AA-3′.

### pMK-IRAK4-mScarlet-I-HDR

5′ and 3′ homology arms were designed from the mouse *Irak4* gene (ENSMUSG00000059883) covering a distance of 702 bp and 722 bp on either side of the TAA stop codon. The 5′ and 3′ homology arms were ordered as gBlocks from Integrated DNA Technologies. mScarlet-I was inserted between these homology arms and fused to the IRAK4 C terminus via a 3×(Gly-Gly-Ser) linker. The following primers were used: mScarlet-I, 5′-GAA​GTG​GAG​GTT​CTG​GTG​GTA​GT-3′ and 5′-CTC​CAG​GTT​CTC​GAG​AAG​TAA​AAA​AAA​AAA​AAA​AAA​AGT​TTA​TTA​GTT​TTA​CTT​GTA​CAG​CTC​GTC​CAT​GCC-3′; and pMK vector backbone, 5′-CAT​GGT​CAT​AGC​TGT​TTC​CTT​GCG-3′ and 5′-GCT​GGC​CGT​CGT​TTT​ACA​ACG-3′.

### pMK-IRAK1-mScarlet-I-2A-PuroR-HDR

5′ and 3′ homology arms were designed from the mouse *Irak1* gene (ENSMUSG00000031392) covering a distance of 2,251 bp and 764 bp on either side of the TGA stop codon. A mScarlet-I-2A-PuroR cassette was inserted between these homology arms and fused to the IRAK1 C terminus via a 3×(Gly-Gly-Ser) linker. The following primers were used: 5′ homology arm, 5′-GTT​ATC​TGT​CAT​TCT​GTT​GGC​TGT​ATG-3′ and 5′-CCC​TGA​GAC​TTT​TCA​GGT​TCT​AAA​TCC​AAG​CC-3′; 3′ homology arm, 5′-TTT​GTT​CAC​TCT​GAC​AAA​TCC​CTC​AG-3′ and 5′-GGC​TTG​CAT​ATA​TCC​ACC​CAA​GAT​G-3′; mScarlet-I-2A-PuroR cassette, 5′-TTA​GAA​CCT​GAA​AAG​TCT​CAG​GGA​CCT​GAA​GAA​AGT​GAT​GAA​TTt​CAG​AGC​GGA​GGA​AGT​GGA​GGT​TCT​GGT​GGT​AGT​GTG-3′ and 5′-GGA​TTT​GTC​AGA​GTG​AAC​AAA​TCA​GGC​ACC​GGG​CTT​GCG​GGT​CAT-3′; and pMK vector backbone, 5′-CAT​CTT​GGG​TGG​ATA​TAT​GCA​AGC​CCA​TGG​TCA​TAG​CTG​TTT​CCT​TG-3′ and 5′-CAT​ACA​GCC​AAC​AGA​ATG​ACA​GAT​AAC​CTG​GCC​GTC​GTT​TTA​CAA​C-3′.

### Generation of CRISPR/Cas9 sgRNA vectors for endogenous labeling of MyD88, IRAK4, and IRAK1

sgRNA targeting ±50 bp of the C terminus stop codon of MyD88, IRAK4, and IRAK1 were designed using the web-based Benchling CRISPR design tool. The following sgRNAs were selected for each target: MyD88, 5′-CCT​GCC​CTG​AAG​ATG​ACC​CT-3′ and 5′-TGA​CAT​ACC​TAG​GGC​TCC​CA3′; and IRAK4, 5′-TTC​AAG​ACA​TCG​GCT​TAA​CC-3′ and IRAK1 5′-TTA​GAA​CCT​GAA​AAG​AGC​CA-3′. We designed complementary oligonucleotides to be ligated into Bbs1-digested *Streptococcus pyogenes* Cas9 and chimeric guide RNA expression plasmid pX330 (pX330-U6-Chimeric_BB-CBh-hSpCas9; Addgene; #42230). sgRNA oligonucleotides were ordered from Integrated DNA Technologies. Complementary sgRNA oligonucleotides were 5′ phosphorylated with T4 polynucleotide kinase, annealed, ligated into Bbs1-digested pX330 using Quick Ligase (NEB). pX330 plasmids were transformed into NEB Stable*–*competent cells. All sgRNA pX330 plasmids were sequence-verified.

### Generation of CRISPR/Cas9 engineered cell lines

EL4 cells were electroporated with pX330 Cas9/gRNA expressing vector and the pMK vector encoding the HDR template with the Neon Transfection System. EL4 cells were electroporated with the following conditions: voltage (1,080 V), width (50 ms), and number of pulses (one). For single editing of the MyD88 gene locus, 1.5 µg total of MyD88 sgRNA-Cas9 and MyD88-GFP HDR template plasmids (in equal molar ratio) were electroporated into 2 × 10^7^ cells/ml for a 10-µl reaction with buffer R according to the manufacturer's protocol. Cells were plated to 24-well plates in RPMI culture medium without antibiotics and expanded for 7 d.

Monoclonal cell lines were generated by FACS. Cells were sorted using a BD FACS Aria II at the Deutsches Rheuma-Forschungszentrum Berlin Flow Cytometry Core Facility. To isolate gene-edited EL4 cells, we performed a bulk sorting of GFP-positive cells (Fig. S1 B). This population was expanded and single cell lines sorted onto 96-well plates containing culture medium with 15% EL4.NOB-1 \–conditioned RPMI medium. The same strategy was applied for double editing of MyD88/IRAK4 or MyD88/IRAK1 gene loci. 1.5 µg of sgRNA-Cas9 and HDR template plasmids (in equal molar ratio) was electroporated simultaneously. For the selection of IRAK1-edited events, 1.5 µg/ml puromycin was added to the cell culture medium 24 h after electroporation. EL4 cells were selected in puromycin for 48 h.

Monoclonal cell lines were verified using diagnostic PCR, Sanger sequencing, Western blot analysis, and microscopy (Fig. S1, A–D; and Fig. S4, B and C). First, genomic DNA was isolated from selected monoclonal cell lines using QuickExtract DNA Extraction Solution (Epicentre). To test for gene editing and positional insertion of the mGFP/mScarlet-I cassette, PCR primers were designed to amplify a DNA fragment that contained the junctions between the mGFP/mScarlet-I open reading frame, the 3′ or 5′ homology arm, and the gene locus. The following primers were used: MyD88, GFP-forward 5′-TGC​CCG​ACA​ACC​ACT​ACC​TGA​GC-3′ and MyD88-reverse 5′-GAA​GAT​GCA​AAC​CTC​GCT​GCT​GGG​G-3′; IRAK4, IRAK4-forward 5′-GAG​CCA​CCT​TTC​AAC​CTT​CCG​T-3′ and mScarlet-reverse 5′-CAC​CTT​CAG​CTT​GGC​GGT​CTG-3′; and IRAK1, mScarlet-forward 5′-GAC​CGC​AAG​TTG​GAC​ATC​ACC​T-3′ and IRAK1-reverse 5′-AGC​CTG​CCT​AGG​CAG​GCA​GGT​AGT​C-3′.

To check single-cell clones for homozygosity, we designed PCR primers that amplified a fragment containing the mGFP/mScarlet-I cassette, the entire 3′ or 5′ homology arms, and the junction between the homology arms and the gene locus (see Fig. S1 C). The following primers were used: MyD88, forward 5′-CTG​GAC​CCG​CCT​TGC​CAA​GGC-3′ and reverse 5′-GAA​GAT​GCA​AAC​CTC​GCT​GCT​GGG​G-3′; IRAK4, forward 5′-GAG​CCA​CCT​TTC​AAC​CTT​CCG​T-3′ and reverse 5′-GCA​CTA​TGC​TAC​CAT​GTT​AAA​CAT​AAA​GCG​C-3′; and IRAK1, forward 5′-CAA​AGT​TCT​GTG​CTC​ATG​GTT​CAT​GTC​AGG​G-3′ and reverse 5′-AGC​CTG​CCT​AGG​CAG​GCA​GGT​AGT​C-3′. PCR products were analyzed on a 0.8–1% agarose gel. Homozygosity was detected by the presence of a single high-molecular-weight DNA band (Fig. S1 C). Gel fragments of homozygous clones were extracted using Monarch Nucleic Acid Purification Kits (NEB) and submitted for Sanger sequencing.

To confirm the presence of mEGFP/mScarlet-I fusion protein of the correct molecular weight, CRISPR/Cas9-edited cell clones were analyzed by Western blot. Lysates were blotted with antibodies specific for the target protein, and then stripped and reprobed with antibodies specific for GFP or mScarlet-I (Fig. S1 D; and Fig. S4, B and C). Primary antibodies include goat polyclonal anti-MyD88 (R&D Systems; #AF3109), rabbit polyclonal anti-IRAK4 (Cell Signaling Technology; #4363), rabbit monoclonal anti-IRAK1 (D51G7; Cell Signaling Technology; #4504), rabbit polyclonal anti-GFP (ChromoTek; #PABG1), mouse monoclonal anti-RFP (ChromoTek; #6g6), and rabbit polyclonal anti-GAPDH (AbFrontier; #LF-PA0018). Finally, all cell clones were checked by microscopy for correct localization of fluorescent signals.

### Generation of CRISPR/Cas9 sgRNA IRAK4 and IRAK1 KO vectors

sgRNAs targeting the first coding exon of the N terminus of IRAK4 and IRAK1 were designed using the web-based Benchling CRISPR design tool. sgRNAs were selected for each target. We selected the following sgRNA sequences: IRAK1, 5′-GCC​TGG​AAC​CAC​AGG​CTC​CCC-3′; and IRAK4, 5′-GAG​GTT​GCG​TAT​GTA​TGT​CGA-3′. We designed complementary oligonucleotides to be ligated into Bbs1-digested *S. pyogenes* Cas9 and chimeric gRNA expression plasmid pX459v2.0 (pX459v2.0-pSpCas9[BB]-2A-Puro; Addgene; #62988) or pX459v2.0-HypaCas9 (pX459v2.0-HypaCas9-2A-Puro; Addgene; #108294). sgRNA oligonucleotides were ordered from Integrated DNA Technologies. Complementary sgRNA oligonucleotides were 5′-phosphorylated with T4 polynucleotide kinase, annealed, and ligated into Bbs1-digested pX459v2.0 using Quick Ligase (NEB). pX330 plasmids were transformed into *NEB Stable*–competent cells. All sgRNA pX459v2.0 plasmids were sequence-verified.

### HDR DNA template design for CRISPR/Cas9 generation of IRAK1 and IRAK4 KO cells

Plasmid DNA repair templates to generate KO cell lines were designed in the same way as described above. The homology arms were assembled with DNA fragments encoding a blasticidin-resistant cassette followed by 3×STOP codons plus 1 nt (to induce a downstream frameshift) into pMK plasmid backbone using Gibson Assembly. All HDR KO template plasmids were sequence-verified. Full details of the HDR DNA template plasmid construction are given below.

### pMK-IRAK4-KO-BlastR-3×Stop-HDRtemp

5′ and 3′ homology arms were designed from the mouse *Irak4* gene (ENSMUSG00000059883) covering a distance of 254 bp and 657 bp on either side of the ATG start codon. A blasticidin-resistant cassette with 3′ 3×STOP codons plus 1 nt was inserted between these homology arms. The following primers were used: 5′ homology arm, 5′-CGT​TGT​AAA​ACG​ACG​GCC​AGT​TCT​AGA​TGC​TGT​CTC​TGA​AAC​TGT​TG-3′ and 5′-GTG​GCG​CAT​GTC​TTT​AAT​GCC-3′; 3′ homology arm, 5′-GTA​ACT​CCT​CCT​CCC​CAT​CAC​A-3′ and 5′-CAA​GGA​AAC​AGC​TAT​GAC​CAT​GGT​TGC​GTT​TTA​CTG​CAG​AAC​AAA​GTA​C-3′; BlastR-3×Stop, 5′-GAT​GGG​GAG​GAG​GAG​TTA​CCT​TAT​ATG​GAA​CTG​ATT​GTA​TCT​GTC​GTC​GCC​GGA​CGG​CTT​TTT​GAT​AGC​TAC​TGC​TAA​TTT​CTT​CCA​CCC​TTC​TTG​AGG​ATC​AAT​AAA​ATC​CGA​CAG​CTT​CCT​AAG​GAT​CCC​CAC​ATT​AAG​GTT​GCG​TAT​GTA​TGT​CGA​TTG​TGT​CAA​CAG​CTT​GTT​GTC​ATC​ATC​AGC​CCT​CCC​ACA​CA-3′ and 5′-CGT​CAC​TTA​GTT​CAT​CAT​CAG​CCC​TCC​CAC​ACA​TAA​CCA​GAG​GGC​AGC​AAT​TCA​CGA​ATC-3′; and pMK vector backbone, 5′-CAT​GGT​CAT​AGC​TGT​TTC​CTT​G-3′ and 5′-CTG​GCC​GTC​GTT​TTA​CAA​CGT​C-3′.

### pMK-IRAK1-KO-BlastR-3×Stop-HDRtemp

5′ and 3′ homology arms were designed from the mouse *Irak1* gene (ENSMUSG00000031392) covering a distance of 896 bp and 874 bp on either side of the ATG start codon. A blasticidin-resistant cassette with 3′ 3×STOP codons plus 1 nt was inserted between these homology arms. The following primers were used: 5′ homology arm, 5′-CAG​TAG​AGG​AAT​AGG​CAA​GAG​ACA​CTC​ATT​CAT​GTG​GCT​AAA​GGT​CAC​AAC​AGG-3′ and 5′-GCG​GCG​GCG​GCG​GCC​ATG-3′; 3′ homology arm, 5′-TGA​TGA​TGA​ACT​AAG​TGA​CGT​ACG​AGG​TGC​CAC​CCT​GGG​TTA​TG-3′ and 5′-CCC​TGG​GGG​TTA​AGA​GAC​ACC​TAC​CTT​GGT​GGA​GG-3′; BlastR-3×Stop, 5′-GGC​GGC​GGC​GGC​GGC​CAT​GGC​CAA​GCC​TTT​GTC​TCA​AGA​AGA​ATC-3′ and 5′-CGT​CAC​TTA​GTT​CAT​CAT​CAG​CCC​TCC​CAC​ACA​TAA​CCA​GAG​GGC​AGC​AAT​TCA​CGA​ATC-3′; and pMK vector backbone, 5′-GTA​GGT​GTC​TCT​TAA​CCC​CCA​GGG​CAT​GGT​CAT​AGC​TGT​TTC​C-3′ and 5′-GTG​TCT​CTT​GCC​TAT​TCC​TCT​ACT​GCT​GGC​CGT​CGT​TTT​ACA​AC-3′.

### Generation of CRISPR/Cas9 IRAK1 and IRAK4 KO cell lines

Two methods were used to generate IRAK1/IRAK4 KO cell lines. In the first method, EL4-MyD88-GFP cells were electroporated with pX459v2.0 Cas9/gRNA. 24 h after electroporation, the cells were selected in puromycin for 3 d. After selection, cells were single-cell sorted into 96-well plates. Isolated clones were then screened (see below for details). We found this method inefficient with many clones still WT. Only one IRAK1 KO clone was isolated using this method (Fig. S5 B, clone 1).

We developed a second more efficient method that used HDR templates to insert a blasticidin cassette followed by 3×STOP codons. In this method, EL4-MyD88-GFP cells were electroporated with the pX459v2.0 Cas9/gRNA and a pMK vector encoding the KO-HDR template with the Neon Transfection System (see above for conditions). Electroporated cells were maintained in complete RPMI medium for 3 d after electroporation. On the fourth day, cells were split into RPMI medium containing blasticidin (6 µg/ml). Cells were selected for 7–14 d with blasticidin and then sorted in 96-well plates to select single-cell clones.

Monoclonal KO cell lines were verified using diagnostic PCR, Sanger sequencing, and Western blot analysis (Fig. S4 C; and Fig. S5, A and B). First, genomic DNA was isolated from selected monoclonal cell lines using QuickExtract DNA Extraction Solution (Epicentre). Primers specific to the blasticidin-resistant cassette and IRAK1/IRAK4 gene loci were used to verify the insertion. We used the following primer sequences: IRAK4, gene loci forward, 5′-TGG​GTC​GGA​GTG​AAA​GCT​GCT​C-3′ and blasticidin reverse, 5′-GCT​GTC​CAT​CAC​TGT​CCT​TCA​CTA​TG-3′; and IRAK1: blasticidin forward 5′-GGA​CAG​TGA​TGG​ACA​GCC​GAC-3′ and gene loci reverse 5′-CTT​CCA​GCA​GTC​AAG​CCC​AGA​GA-3′.

We designed a second set of PCR primers that amplified a fragment containing the blasticidin cassette, the entire 3′ or 5′ homology arms, and the junction between the homology arms and the gene locus. We used the following primer sequences: IRAK4, forward 5′-TGG​GTC​GGA​GTG​AAA​GCT​GCT​C-3′ and reverse 5′-GAC​ACT​TGC​TGG​AAG​GTC​AAT​ATG​G-3′; and IRAK1, forward 5′-AGG​CCG​CGG​AGG​GCA​AGA​TG-3′ and reverse 5′-GGA​AAC​AGG​GAG​TGG​AAC​CTG​GA-3′. PCR products were analyzed on a 0.8–1% agarose gel, and gel fragments of clones were extracted using Monarch Nucleic Acid Purification Kits (NEB) and submitted for Sanger sequencing. We found only homozygous blasticidin insertion clones for the KO of IRAK1. In contrast, with IRAK4, we found only heterozygous clones; however, sequencing confirmed the presence of an insertion in the second allele resulting in a frameshift. Western blot analysis confirmed all clones to be KO for IRAK4 or IRAK1 (Fig. S5, A and B). Western blot analysis was performed with the following antibodies: goat polyclonal anti-MyD88 (R&D Systems; #AF3109), rabbit polyclonal anti-IRAK4 (Cell Signaling Technology; #4363), rabbit monoclonal anti-IRAK1 (D51G7; Cell Signaling Technology; #4504), rabbit polyclonal anti-GFP (ChromoTek; #PABG1), mouse monoclonal anti-RFP (ChromoTek; #6G6), and rabbit polyclonal anti-GAPDH (AbFrontier; #LF-PA0018).

### Generation of lentiviral pHR-dSV-IRAK4-mScarlet construct

To construct pHR-dSV-IRAK4-mScarlet fusion, mouse IRAK4 (BC051676) was ordered as a gBlock (Integrated DNA Technologies). IRAK4 was fused to mScarlet-I via a 3×GGS linker. DNA fragments were assembled with pHR-dSV lentiviral plasmid digested with Mlu1/Not1 using Gibson Assembly.

### Lentiviral production and generation of stable expressing EL4 cell lines

Lentivirus particles were produced in HEK-293T cells by cotransfection of the pHR transfer plasmids with second-generation packaging plasmids pMD2.G and psPAX2 (a gift from Didier Trono, École Polytechnique Fédérale de Lausanne, Lausanne, Switzerland; Addgene; #12259 and #12260). Virus particles were harvested from the supernatant after 48–72 h, filtered, and applied to EL4 cells. After 3 d, the cells were resuspended in fresh RPMI media. After 1 wk, FACS was performed to select cell populations with homogenous IRAK4-mScarlett expression levels.

### Assay of IL2 release in WT and gene-edited EL4 cells

To measure IL2 release, we used the Mouse IL-2 DuoSet ELISA kit (R&D Systems; DY402-05) following the manufacturer’s protocol. Briefly, 10^6^ cells in 150 µl medium per well were seeded into a 48-well plate. Cells were allowed to settle for 30 min before being stimulated with IL1β in 50 µl medium per well at a final concentration of 10 ng/ml. For the unstimulated control, 50 µl medium was added. After 24 h, plates were centrifuged (300 *g* for 5 min), and supernatants were transferred into a new plate. Supernatants were stored at −80°C until IL2-ELISA analysis. Absorbance readings were acquired on a VersaMax Microplate Reader (Molecular Devices) at 450 nm. IL2 release was assayed on 3 independent days in triplicate. The obtained results were normalized based on the EL4 WT IL2 release.

### Imaging chambers and SLBs

SLBs were prepared using a previously published method (Taylor et al., 2017). Phospholipid mixtures consisting of 97.5% mol 1-palmitoyl-2-oleoyl-*sn*-glycero-3-phosphocholine, 2% mol 1,2-dioleoyl-sn-glycero-3-[(N-(5-amino-1-carboxypentyl)iminodiacetic acid)succinyl] (ammonium salt), and 0.5% mol 1,2-dioleoyl-sn-glycero-3-phosphoethanolamine-N-[methoxy(polyethylene glycol)-5000] were mixed in glass round-bottom flasks and dried down with a rotary evaporator. All lipids used were purchased from Avanti Polar Lipids. Dried lipids were placed under vacuum for 2 h to remove trace chloroform and resuspended in PBS. Small unilamellar vesicles were produced by several freeze–thaw cycles. Once the suspension had cleared, the lipids were spun in a benchtop ultracentrifuge at 35,000 *g* for 45 min and kept at 4°C for up to 5 d.

SLBs were formed in 96-well glass-bottom plates (MatriCal) or set up on coverslips (25 mm diameter; Marienfeld-Superior; no. 1.5 H) mounted in an Attofluor chamber (Thermo Fisher Scientific). 96-well plates were cleaned for 30 min with a 5% Hellmanex solution containing 10% isopropanol heated to 50°C, then incubated with 5% Hellmanex solution for 1 h at 50°C, followed by extensive washing with pure water. 96-well plates were dried with gas nitrogen and sealed until needed. To prepare SLB on 96-well plates, individual wells were cut out and base-etched for 15 min with 5 M KOH and then washed with water and finally PBS. Coverslips were washed in acetone and ethanol in an ultrasonic cleaner before rinsing extensively in water. Coverslips were then cleaned with a solution of KOH and hydrogen peroxide for 10 min, followed by extensive washing in water. Finally, coverslips were cleaned with a solution of 6% HCl (vol/vol) and 6.3% (vol/vol) hydrogen peroxide. Cleaned coverslips were stored in water before being used for SLBs formation and microscopy.

To form SLBs, small unilamellar vesicle suspensions were deposited in each well or coverslip and allowed to form for 1 h. We found that small unilamellar vesicle suspensions containing 0.5% mol 1,2-dioleoyl-sn-glycero-3-phosphoethanolamine-N-[methoxy(polyethylene glycol)-5000] formed best at 45°C. After 1 h, wells were washed extensively with PBS. SLBs were incubated for 15 min with Hepes-buffered saline (HBS; 20 mM Hepes, 135 mM NaCl, 4 mM KCl, 10 mM glucose, 1 mM CaCl_2_, and 0.5 mM MgCl_2_) with 5 mM NiCl_2_ to charge the 1,2-dioleoyl-sn-glycero-3-[(N-(5-amino-1-carboxypentyl)iminodiacetic acid)succinyl] lipid with nickel. The SLBs were then washed in HBS containing 0.1% BSA to block the surface and minimize nonspecific protein adsorption. For SLBs set up on 96-well plates, the total well volume was 625 µl (manufacturer’s specifications). Each well was completely filled with HBS containing 0.1% BSA, and 525 µl was removed, leaving 100 µl in each well. After blocking, the SLBs were functionalized by incubation for 1 h with 100 µl His-tagged proteins. The labeling solution was then washed out with HBS.

### Protein expression, purification, and labeling

To functionalize membranes with active mouse IL1β, we created a protein linker that could tether the mature IL1β cytokine to SLBs. To aid in solubility and expression, we designed this tether not to be directly fused to mature IL1β on the same peptide chain. We used a SpycatcherV2 domain to covalently link this tether to recombinant mature mouse IL1β expressed with a C terminus SpytagV2 peptide (AHIVMVDAYKPTK). Spycatcher is an engineered protein domain derived from the *S. pyogenes* CnaB2 domain that is able to form an isopeptide bond to the SpyTag peptide (Keeble et al., 2017). To construct this protein tether, we created a codon-optimized HaloTag and SpycatcherV2 open reading frames and ordered them as gBlocks from Integrated DNA Technologies. To enhance solubility and expression of this fusion protein, the HaloTag and Spycatcher open reading frames were separated by a Tencon domain (a high-stability FNIII domain designed through multiple-sequence alignment; Jacobs et al., 2012). Using Gibson assembly, gene fragments were cloned into a pET28a vector containing an N-terminal 10×His tag (pET28a-His10-Halo-Tencon-SpycatcherV2). We also created an identical version where mScarlet was substituted for HaloTag (pET28a-His10-mScarlet-I-Tencon-SpycatcherV2). The mature active form of mouse IL1β (aa 118–169) was codon-optimized for *Escherichia coli* expression and ordered as a gBlock from Integrated DNA Technologies. The gBlock was designed to contain a C-terminal SpyTagV2 connected via a 13-aa glycine–serine linker. We used Gibson assembly to clone this fragment into pET28a (pET28a-MmIL1β-Spytag).

All proteins were expressed in BL21-DE3 Rosetta *E. coli* (Novagen). The bacterial cell pellets were resuspended in the lysis buffer (20 mM Hepes and 150 mM NaCl with protease inhibitors) and lysed using a French press. To covalently couple His10-Halo/mScarlet-I-Tencon-Spycatcher to MmIL1β-Spytag, the cleared lysates were mixed and incubated with mild agitation for 1 h at 4°C. To ensure complete Spycatcher-Spytag conjugation, the lysates were mixed in a 2:1 ratio (vol:vol, based on starting bacterial culture volume) of MmIL1β-Spytag to His10-Halo/mScarlet-I-Tencon-Spycatcher. After the conjugation, the His10-Halo/mScarlet-I-Tencon-Spycatcher-IL1β-Spytag was purified by Ni-NTA resin. Conjugation was monitored by mobility shift using SDS-PAGE. After elution, the protein was dialysed into 20 mM Hepes overnight, followed by anion exchange chromatography on a MonoQ column. This was followed by gel filtration over Superdex 200 26/600 into storage buffer (20 mM Hepes and 150 mM NaCl). The protein was snap-frozen with the addition of 20% glycerol in liquid nitrogen and placed at −80°C for long-term storage. In text, these proteins are referred to as His10-mScarlet-IL1β or His10-Halo-IL1β.

Following purification, the His10-Halo-Tencon-Spycatcher-IL1β-Spytag protein was either snap-frozen and stored at –80°C or directly used for HaloTag labeling. To label the HaloTag, a 2.5× molar excess of JF646-HaloLigand was mixed with the protein and incubated at room temperature for 1 h followed by an overnight incubation at 4°C. After labeling, the protein was gel-filtered over a Superdex 200 26/600 into storage buffer and snap-frozen with the addition of 20% glycerol in liquid nitrogen and placed in −80°C for storage. The degree of labeling was calculated with a spectrophotometer by comparing 280-nm and 640-nm absorbance (usually 85–95% labeling efficiency was achieved).

### Immunofluorescence confocal microscopy analysis of RelA and phospho-p38 localization

To analyze the nuclear localization of RelA or phospho-p38 levels in IL1β-stimulated EL4 cells, SLBs were labeled with fluorescent IL1β, and unlabeled SLBs served as unstimulated controls. On the day of an experiment, EL4.NOB1 cells endogenously expressing MyD88-GFP were transferred to serum-free media and incubated for 3–4 h. Then, 10^6^ EL4 cells were applied to each supported membrane. Cells were then incubated at 37°C for 30–45 min before the addition of an equal well volume of 2× fixative (7% wt/vol PFA with 0.1% wt/vol Triton X-100). Cells were fixed for 20 min at room temperature, followed by incubation with a final concentration of 30 mM glycine for 10 min at room temperature to quench PFA. Cells were washed with PBS, then blocked in PBS 10% (wt/vol) BSA containing 4% normal goat serum for 1 h at room temperature or overnight at 4°C before addition of primary antibody. Fixed cells were labeled overnight with primary antibodies diluted in PBS 10% (wt/vol) BSA containing 0.1% Triton X-100 (final concentration for anti-RelA: rabbit monoclonal; 1:400; Cell Signaling Technology, #8242; for phospho-p38: rabbit monoclonal; 1:1,600; Cell Signaling Technology; #4511). The next day, cells were washed five times with PBS and labeled with secondary antibodies (goat anti-rabbit conjugated to Alexa Fluor 555/647; 1:1,000; Invitrogen; #A21428/A21246) and FluoTag-X4 anti-GFP conjugated to Atto488 (1:500; Nano Tag Biotechnology; #N0304-At488-L to boost the MyD88-GFP signal) for 1 h at room temperature or overnight at 4°C. Cells were then labeled with DAPI at room temperature. Finally, cells were washed again five times in PBS.

For confocal imaging, mounted coverslips of stimulated and unstimulated EL4 cells were imaged on a Zeiss Airyscan LSM 880 using a Plan Apo 63× 1.4 NA oil-immersion objective and controlled by Zeiss ZEN software. Fields of view containing multiple cells were selected based on the DAPI and MyD88-GFP channels. Z-stacks of the entire cellular volume were acquired in the DAPI, GFP, and Alexa Fluor 647 channels. The nuclear staining intensity of RelA and phospho-p38 was analyzed in Fiji. Z-stacks of the DAPI, GFP, and Alexa Fluor 647 channels were imported into Fiji. Using the DAPI channel, three Z-planes were selected, and a maximum projection of the Z-planes was used to create a new 32-bit image. Maximum projection images were created of the identical Z-planes for the Alexa Fluor 647 channel. The DAPI channel was segmented to identify cell nuclei. The detected nuclear boundaries were used to extract nuclear staining intensity from the Alexa Fluor 647 channel (e.g., the RelA or phospho-p38 staining intensity; Fig. 1 E and Fig. S1 G). Reconstructed axial views of cells shown in Fig. 1 D and Fig. S1 F were generated in Fiji.

### TIRF microscopy data acquisition

Imaging of MyD88-GFP and IRAK recruitment was performed on an inverted microscope (Nikon TiE) equipped with a Nikon fiber launch TIRF illuminator. Illumination was controlled with a laser combiner using the 488-, 561-, and 640-nm laser lines at ∼0.35, ∼0.25, and ∼0.17 mW laser power, respectively (laser power measured after the objective). Fluorescence emission was collected through filters for GFP (525 ± 25 nm), RFP (595 ± 25 nm), and JF646 (700 ± 75 nm). All images were collected using a Nikon Plan Apo 100× 1.4 NA oil-immersion objective that projected onto a Photometrics 95B Prime sCMOS camera with 2 × 2 binning (calculated pixel size of 150 nm) and a 1.5× magnifying lens. Image acquisition was performed using NIS-Elements software. All experiments were performed at 37°C. The microscope stage temperature was maintained using an OKO Labs heated microscope enclosure. Images were acquired between intervals of 1–5 s using exposure times of 60–100 ms.

### Imaging EL4 cells endogenously expressing MyD88-GFP, IRAK4-mScarlet, or IRAK1-mScarlet on IL1β functionalized SLBs with TIRF microscopy

His10-Halo-JF646-IL1β–functionalized SLBs were set up as described above. To quantify the density of IL1β on the SLB, wells were prepared that were functionalized with identical labeling protein concentration and time, but with different molar ratios of labeled to unlabeled His10-Halo-IL1β. Before application of cells, SLBs were analyzed by TIRF microscopy to check formation, mobility, and uniformity. Short time series were collected at wells containing a ratio of labeled to unlabeled His10-Halo-IL1β (e.g., <1 His10-Halo-JF646-IL1β molecule/µm^2^) to calculate ligand densities on the SLB based upon direct single molecule counting. All experiments were performed at IL1 SLB densities of 10–30 molecules/µm^2^.

Before each imaging experiment, we acquired calibration images using recombinant mEGFP. To image a single GFP fluorophore, recombinant purified monomeric EGFP was diluted in HBS and adsorbed to KOH-cleaned glass. Single molecules of GFP were imaged using identical microscope acquisition settings to those used for cellular imaging. To image live cells, EL4 cells were pipetted onto supported lipids bilayers functionalized with His10-Halo-JF646-IL1β. EL4 cells expressing only MyD88-GFP were sequentially illuminated for 60–100 ms with 488 nm at a frame interval of 1 s (Fig. 3). EL4 cells expressing MyD88-GFP, IRAK4-mScarlet, or IRAK1-mScarlet were sequentially illuminated for 60–100 ms with 488-nm and 100 ms with 561-nm laser line at a frame interval of 1 s (Fig. 4). Diffraction-limited punctate structures of MyD88-GFP, IRAK4-mScarlet, or IRAK1-mScarlet were detected and tracked using the Fiji TrackMate plugin (Tinevez et al., 2017). For experiments with IRAK4 inhibitor (PF-06650833; Sigma-Aldrich; #PZ0327), EL4 was incubated for 30 min at 20 µM before imaging in the presence of the inhibitor. Control cells in IRAK4 inhibitor experiments were imaged in medium containing 0.1% DMSO (vol/vol).

### FRAP experiments and data analysis

FRAP experiments were performed at the Advanced Medical BioImaging Core Facility at the Universitätsmedizin Berlin on a Nikon TIRF microscope (Nikon Eclipse Ti-E) with the FRAPPA module controlled with NIS-Elements software. Fluorescent images were acquired with a Nikon Plan Apo 100× 1.4 NA oil-immersion objective and projected on a Photometric Prime 95B sCMOS camera. All other imaging conditions were identical to those described above. For FRAP analysis of Myddosomes in gene-edited EL4 cells, we prepared SLBs labeled with either IL1β-mScarlet-His10 or IL1β-Halo-JF646-His10, depending on whether the photobleach experiments were performed in EL4-MyD88-GFP or EL4-MyD88-GFP/IRAK4-mScarlet and EL4-MyD88-GFP/IRAK1-mScarlet gene-edited cell lines. Before the addition of cells to the imaging chamber, we analyzed SLB formation and mobility by visual inspection of the fluorescently labeled IL1β. We prepared EL4 cells for imaging by washing in PBS and resuspending in HBS at a final concentration of 10^5^ cells/ml. To ensure FRAP analysis of fully assembled Myddosomes, we incubated 10^4^ EL4 cells with IL1β-functionalized SLBs for at least 15 min before image acquisition. After incubation, we selected EL4 cells containing multiple MyD88-GFP–labeled Myddosomes for FRAP analysis. The photobleach spot was centered on large stationary Myddosomes or in some cases a cluster of Myddosomes. Images were recorded 10 s before and 60 s after photobleaching at a time interval of one image per second. Photobleaching experiments on super MyD88 oligomers in IRAK4 KO cells were performed identically to those described above with the following differences. First, cells were incubated with SLBs for ∼5 min or 1 h before image acquisition. All images were acquired with a time interval of 2 s. Second, for IRAK4 KO cells incubated for 5 min, oligomers were photobleached three times with a 5-min interval between bleaching. For those incubated for 1 h, images were acquired 120 s after photobleaching.

To quantify FRAP recovery, we adapted the approach described by Kang et al. (2015). We determined the integrated intensity of the photobleached region as a function of time. The background intensity was measured from a neighboring region to the photobleached spot and was subtracted from all time points. The data were normalized to the prebleach intensity using the following equation: $Intensity{\left(t\right)}_{normalized}=\frac{Intensity(t)-Intensity(0)}{Intensity(pre\;bleach)-Intensity(0)},$ where *Intensity(prebleach)* is the average intensity preceding photobleaching, and *Intensity(0)* is the intensity immediately after photobleaching. Measurements from multiple photobleached Myddosomes were averaged, and the SD was calculated (Fig. 5, D–F). We conducted three independent experimental replicates on different days for each FRAP experiment (e.g., MyD88-GFP, IRAK1-mScarlet, and IRAK4-mScarlet). To ensure intensity measurements were only recorded from single MyD88 oligomers in IRAK4 KO cells, we manually tracked the oligomer before and after photobleaching. This was only possible with MyD88 super-oligomer bleached after a 5-min incubation interval (Fig. 7 A) due to the lack of recovery after 1 h (Fig. 7 B).

### Quantification and statistical analysis

All data are expressed as mean ± SD or mean ± SEM as stated in the figure legends and Results. The exact value of *n* and what *n* represents (e.g., number of cells, MyD88-GFP puncta, or experimental replicates) is stated in the figure legends and Results. Means of experimental replicates were compared using an unpaired two-tailed Student’s *t* test implemented in R studio. Data distribution was assumed to be normal based on density plots, but this was not formally tested. Data and scripts used in this study are available at https://doi.org/10.5281/zenodo.4012312 and https://github.com/MJ-Taylor-Lab/myddosome-dynamics-pipeline. Given large file sizes of the RelA and phospho-p38 staining data presented in Fig. S2, A and C; and Fig. 3 G, this raw data is available from the lead contact upon request.

#### Quantification and analysis of MyD88-GFP intensity, lifetime, and dynamics

To quantify the dynamics of MyD88-GFP assemblies in EL4 cells, we created an image analysis pipeline that ran in Fiji, MATLAB, and R. The MyD88-GFP fluorescence channel images were processed in Fiji to remove background intensity using custom-written macros. First, we subtracted a dark frame image (i.e., an image containing only intensity values from current and noise generated by the camera electronics) from each image file. The dark frame image was created by averaging 5,000 camera images captured without light exposure and with the same shutter speed as the images. The MyD88-GFP channel images were processed with a median filter (radius, 25 pixels) to create an image of the cytosolic background intensity. The resulting median-filtered image of the background intensity was subtracted from the dark frame–subtracted image stack to create a new MyD88-GFP image stack. We quantified the MyD88-GFP signal intensity using this background-subtracted image stack.

Individual cells were identified in the MyD88-GFP fluorescence channel using either a marker-controlled watershed segmentation (implemented with the MorphoLibJ ImageJ plugin; Legland et al., 2016; using a maximum projection of the MyD88-GFP fluorescence channel) or manually. We used the Fiji TrackMate plugin to track the MyD88-GFP particles within each segmented cell. Tracking coordinates generated by TrackMate were imported into MATLAB, and the fluorescence intensity of MyD88-GFP puncta was measured from a 3 × 3 pixel region. To compute the distribution of single fluorophore intensities, images of single mGFP fluorophores absorbed to glass were processed and analyzed identically to MyD88-GFP images. After background subtraction and particle tracking, subsequent analysis was performed in R. We restricted the analysis to MyD88-GFP puncta tracked for three or more frames, to focus our analysis to bona fide MyD88 assemblies nucleating at the plasma membrane.

To analyze the size distribution and stoichiometry of MyD88 multimers, we identified the fluorescence intensity maxima for each tracked MyD88-GFP puncta (Fig. 3 B and Fig. S3 A). To quantify the size (e.g., the copy number of MyD88-GFPs in a tracked puncta), we divided the fluorescent intensities by the mean intensity of single mEGFP fluorophores (measured before each experiment using the same acquisition setting as cellular data; see above). Structural studies have identified 6–8 MyD88 monomers in purified Myddosome complexes (Lin et al., 2010). We reasoned that brighter MyD88-GFP diffraction-limited puncta corresponded to larger multimers of MyD88 and therefore fully assembled Myddosomes. To identify large multimers of MyD88, we estimated the fluorescent intensity distribution for diffraction-limited particles containing 6× GFP. We estimated the fluorescent distribution of 6× GFP to be Gaussian with a mean and variance equal to 6× single GFP fluorophores. Therefore, we used a threshold of ≥4.5× GFP to exclude tracks that consist of MyD88 monomers, dimers, or trimers. We calculated this threshold would select >98% of MyD88-GFP tracks containing 6× MyD88-GFP (although smaller assemblies of 4× and 5× would also be selected). We used this intensity threshold to categorize MyD88-GFP puncta as small (e.g., <4.5× GFP) or large (≥4.5× GFP) MyD88 oligomers. We performed data visualization (e.g., of density plots of intensity maxima distribution and percentage of categorized MyD88 puncta per cells) using ggplot, a data visualization package for R.

To examine the growth of tracked MyD88 puncta, we calculated the maximal growth by subtracting the maximal intensity from the starting intensity (Fig. 3 F). The change in intensity was normalized by dividing it by the intensity of mEGFP. We performed this analysis on MyD88-GFP tracks that had an initial fluorescent intensity <2.5× the mean intensity of GFP. Manual inspection of the date revealed that this cutoff restricted the analysis to nucleating assemblies of MyD88-GFP, and removed those that bud or split from preexisting assemblies where the time point of nucleation could not be accurately determined (Fig. S2, F and G). We tested correlation between ΔIntensity and lifetime using Spearman’s rank correlation analysis. The correlation coefficient (*R*) is reported in the figure legends and text. A 2D histogram of ΔIntensity norm versus lifetime was plotted using ggplot (Fig. 3 F and Fig. S3 E).

#### Analysis of IRAK4 and IRAK1 colocalization with MyD88

In data acquired with EL4 cells expressing MyD88-GFP and IRAK4/IRAK1-mScarlet, the IRAK4-mScarlet and IRAK1-mScarlet fluorescent channel was processed using an identical image processing pipeline that was applied to the MyD88-GFP channel (e.g., background subtraction; see above). The IRAK4 and IRAK1 mScarlet was broken up into minstacks of individual cells. We used the Fiji TrackMate plugin to identify and track IRAK4/1 fluorescent puncta. To quantify colocalization between the MyD88-GFP and IRAK4/1-mScarlet fluorescent channels, we imported the tracking coordinates generated by TrackMate into MATLAB. Using these coordinates, we identified MyD88 and IRAK4/1 particles that colocalized based on two or more consecutive frames where the tracked coordinates were ≤0.25 µm apart. Using these criteria, MyD88 tracked puncta were classified as either positive or negative for IRAK4/1 colocalization.

#### Automated image acquisition, analysis, and quantification of phospho-p38 and RelA staining

SLBs were set up using 96-well plates, as described above. SLBs were set up, labeled with or without His10-IL1β. EL4 cell lines (WT and gene-edited cells lines) were transferred to serum-free media and incubated for 3 h. 100 µl of cells (corresponding to 2 × 10^5^ cells per well) were then applied to 96-well plates (total well volume after addition of cell was 200 µl). Cells were then incubated at 37°C for 30 min, followed by fixation with 200 µl of 2× fixative (7% [wt/vol] PFA with 0.1% [wt/vol] Triton X-100) for 20 min at room temperature. Cells were then incubated with a final concentration of 30 mM glycine for 10 min at room temperature to quench PFA. Cells were washed with PBS, then blocked in PBS 10% (wt/vol) BSA containing 4% normal goat serum for 1 h at room temperature or overnight at 4°C before addition of primary antibody. Fixed cells were labeled overnight at 4°C with primary antibodies diluted in PBS 10% (wt/vol) BSA containing 0.1% Triton X-100 (for anti-RelA: rabbit monoclonal; 1:400; Cell Signaling Technology; #8242; for phospho-p38: rabbit monoclonal; 1:1,600; Cell Signaling Technology; #4511). The next day, cells were washed five times in PBS, and labeled with goat anti-rabbit conjugated to Alexa Fluor 555/647 (1:1,000; Invitrogen; #A21428/A21246,) for 1 h. Cells were then labeled with DAPI for 10 min and 488-phalloidin for 30 min at room temperature. Finally, cells were washed again five times in PBS.

96-well plates were imaged on an inverted microscope (Nikon TiE) equipped with Lumencor Spectra-X illumination. Fluorescent images were acquired with Nikon Plan Apo 20× 0.75 NA air objective lens and projected onto a Photometric Prime 95B camera (pixel width/height of 425 nm) and a 1.5× magnification lens. The fluorescent emission was collected through filters for DAPI (440 ± 40 nm), EGFP (525 ± 30 nm), and Alexa Fluor 555 (595 ± 25 nm)/Alexa Fluor 647 (700 ± 75 nm). Image acquisition was performed using NIS Elements software. Each immune-stained well of a 96-well plate was imaged by setting up a 13 × 13 image grid using the multi-positional function in NIS Elements.

Image processing was done using Fiji macros running from RStudio terminals with parameters dictated by R. First, the dark-frame image (i.e., camera noise with closed shutter) was subtracted from the cell images. All images from the same fluorescent emission channel were averaged. This image was processed with a median-blur filter (100-pixel radius) to create an image of the illumination function of the microscope. Each image was then divided by this illumination image to correct for illumination defects. Background fluorescence was estimated by processing each image with a median blur filter (25-pixel radius). The median filtered image was subtracted from each illumination-corrected image to remove background intensity. We performed subsequent cell segmentation and intensity quantification on background-subtracted and illumination-corrected images.

To identify the nuclear and cytoplasmic area of fixed cells, we used Fiji’s MorphoLibJ marker-controlled watershed segmentation. We segmented the DAPI channel to identify the nuclear area. To identify the cytoplasmic area, we segmented the MyD88-GFP channel (or phalloidin Alexa Fluor 488 for EL4-WT cells) and subtracted the nuclear area. To remove cellular debris and fragments, segmented nuclei had to have a radius 5.5–10 µm, and the cytoplasm had to have a radius <15 µm. Cells had to conform to a certain circularity, and we discarded cells with high eccentricities (1.66 for the nucleus and 2.25 for the cytosol, calculated as major/minor axis of a fitted ellipse). Manual examination of the data revealed these elliptical cells were clumps of cells that incorrectly segmented. To further remove cell aggregates, we stipulated that no more than five cells were allowed within a 16-µm radius of the nuclear centroid. We discarded cells located near the edges of the image. Up until this point, RelA- and phospho-p38–staining images were processed identically.

#### Phospho-p38 image analysis and quantification

Using the segmented nuclear area, the mean staining intensity was extracted from the phospho-p38 image channel. To be able to compare measurements of different replicates and days, the mean intensity of each nucleus was normalized to the median staining intensity of the unstimulated cells of a particular day. We then calculated the experimental replicate (i.e., 1 well of a 96-well plate) means per cell line and condition (stimulated and unstimulated). In general, 10,000 cells were analyzed per well. Mean averages from experimental replicates across cell lines were compared using Student’s *t* test. Data visualization was performed with ggplot and R.

#### RelA nuclear translocation image analysis and quantification

Nuclear translocation was measured by computing the ratio of nuclear to cytoplasmic RelA. We then calculated the experimental replicate (i.e., 1 well of a 96-well plate) means per cell line and condition (stimulated and unstimulated). On average, 5,000 cells per well were analyzed. Cell line means were compared using Student’s *t* test (e.g., stimulated WT replicates were compared with stimulated IRAK 4 KO replicates). Data visualization was done with ggplot (*R*).

### Online supplemental material

Fig. S1 shows the CRISPR/Cas9 gene GFP tagging strategy and validation of the MyD88 editing at the MyD88 gene locus and validation of the IL1 functionalized SLBs system (relevant to Fig. 1). Fig. S2shows validation that tagging Myddosome components does not inhibit IL1R signaling and MyD88 puncta dynamics (relevant to Fig. 2). Fig. S3 shows experimental replicate data and TIRF images (relevant to Fig. 3). Fig. S4 shows validation CRISPR/Cas9 gene tagging of IRAK4 and IRAK1 gene locus with mScarlet and experimental replicate data (relevant to Fig. 4). Fig. S5 shows validation of CRISPR/Cas9 IRAK4/1 KO cell lines and analysis of MyD88 puncta size and lifetime in IRAK4/1 KO cell lines and cells treated with the IRAK4 kinase inhibitor (relevant to Fig. 6). Fig. S6 shows full-length Western blots from Fig. S1 D. Fig. S7 shows full-length Western blots from Fig. S4 B. Fig. S8 shows full-length Western blots from Fig. S4 C. Note Western blot images have been reflected around its vertical axis for Fig. S4 C to enhance clarity. Fig. S9 shows full-length blots for Fig. S6, A and B, for IRAK4 and IRAK1 KO cells. Video 1 shows that IL1β tethered to a supported lipid membrane forms clusters that recruit MyD88-GFP (related to Fig. 2). Video 2 shows single-cell analysis of MyD88-GFP puncta dynamics (related to Fig. 3). Video 3 shows IRAK4 recruitment to clusters of MyD88 (related to Fig. 4). Video 4 shows IRAK1 recruitment to clusters of MyD88 (related to Fig. 4). Video 5 shows FRAP analysis of MyD88-GFP (related to Fig. 5). Video 6 shows MyD88-GFP dynamics in WT, IRAK4 KO, and IRAK1 KO EL4 cells (related to Fig. 6).
